# TREM2 deficiency impairs the energy metabolism of Schwann cells and exacerbates peripheral neurological deficits

**DOI:** 10.1038/s41419-024-06579-9

**Published:** 2024-03-07

**Authors:** Nannan Zhang, Qingjie Ji, Yunfeng Chen, Xiwu Wen, Fangzhen Shan

**Affiliations:** 1 Medical Research Centre, Affiliated Hospital of Jining Medical University, Jining, Shandong China; 2 Department of Respiratory and Critical Care, Affiliated Hospital of Jining Medical University, Jining, Shandong China; 3 Department of Rehabilitation, Affiliated Hospital of Jining Medical University, Jining, Shandong China

**Keywords:** Cell death, Cell growth

## Abstract

Triggering receptor expressed on myeloid cells-2 (TREM2) has been implicated in susceptibility to neurodegenerative disease. Schwann cells (SCs), the predominant glial cell type in the peripheral nervous system (PNS), play a crucial role in myelination, providing trophic support for neurons and nerve regeneration. However, the function of TREM2 in SCs has not been fully elucidated. Here, we found that TREM2 is expressed in SCs but not in neurons in the PNS. TREM2 deficiency leads to disruption of glycolytic flux and oxidative metabolism in SCs, impairing cell proliferation. The energy crisis caused by TREM2 deficiency triggers mitochondrial damage and autophagy by activating AMPK and impairing PI3K-AKT-mTOR signaling. Combined metabolomic analysis demonstrated that energic substrates and energy metabolic pathways were significantly impaired in TREM2-deficient SCs. Moreover, TREM2 deficiency impairs energy metabolism and axonal growth in sciatic nerve, accompanied by exacerbation of neurological deficits and suppression of nerve regeneration in a mouse model of acute motor axonal neuropathy. These results indicate that TREM2 is a critical regulator of energy metabolism in SCs and exerts neuroprotective effects on peripheral neuropathy.

**Figure:**
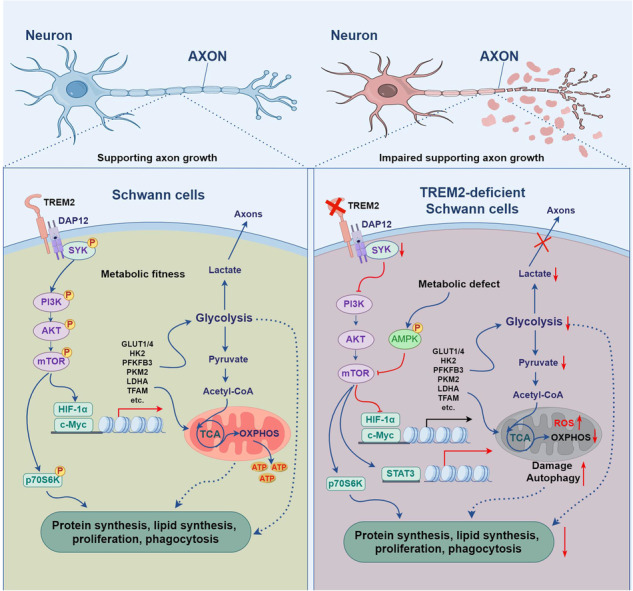
TREM2 deficiency impairs glycolysis and oxidative metabolism in Schwann cells, resulting in compromised cell proliferation. The energy crisis caused by TREM2 deficiency induces mitochondrial damage and autophagy by activating AMPK and impairing PI3K-AKT-mTOR signaling. Moreover, TREM2 deficiency disrupts the energy metabolism of the sciatic nerve and impairs support for axonal regeneration, accompanied by exacerbation of neurological deficits and suppression of nerve regeneration in a mouse model of acute motor axonal neuropathy (by FigDraw).

TREM2 deficiency impairs glycolysis and oxidative metabolism in Schwann cells, resulting in compromised cell proliferation. The energy crisis caused by TREM2 deficiency induces mitochondrial damage and autophagy by activating AMPK and impairing PI3K-AKT-mTOR signaling. Moreover, TREM2 deficiency disrupts the energy metabolism of the sciatic nerve and impairs support for axonal regeneration, accompanied by exacerbation of neurological deficits and suppression of nerve regeneration in a mouse model of acute motor axonal neuropathy (by FigDraw).

## Introduction

Triggering receptor expressed on myeloid cells-2 (TREM2) is a membrane protein expressed primarily on immune cells, especially microglia in the central nervous system (CNS). It plays an essential role in the proliferation, migration and phagocytosis of microglia [1]. In recent years, TREM2 has garnered a great deal of attention due to its association with multiple neurodegenerative disorders, particularly Alzheimer’s disease [2, 3]. TREM2 binds to the transmembrane adaptor DNAX-activating protein of 12 kD (DAP12), which then recruits spleen tyrosine kinase (SYK) via the cytoplasmic immunoreceptor tyrosine-based activation motif, triggering a series of signaling pathways that affect the function of immune cells [4, 5]. Modulators or agonists/antagonists capable of enhancing or inhibiting TREM2 activity are being developed to modulate the immune response and potentially slow disease progression [6–8]. Targeting TREM2 signaling pathway has emerged as a potential therapeutic strategy for treating neurodegenerative and inflammatory diseases.

Recent studies have elucidated the dual functionality of TREM2 within the nervous system. In multiple sclerosis models, TREM2 activation on microglia facilitates myelin debris clearance and remyelination, representing a novel therapeutic approach for demyelinating diseases [9, 10]. Conversely, TREM2/DAP12 signaling in microglia in the spinal dorsal horn elicits a proinflammatory response and exacerbates neuropathic pain, indicating that suppressing TREM2-mediated signaling could be a therapeutic target for treating neuropathic pain [11, 12]. Furthermore, several studies have indicated the role of TREM2 in modulating the immune response in the peripheral nervous system (PNS), impacting the development and progression of certain neuropathies. Kobayashi et al. reported that TREM2 deficiency attenuates microglial neurotoxicity in a mouse model of hypoglossal nerve injury, suggesting that TREM2/DAP12-mediated microglial neuroinflammation exacerbates the degeneration of injured neurons [13]. Zhou et al. demonstrated that TREM2 serves as an innate immune receptor exclusively expressed on microglia in the retina and is responsible for microglial infiltration and phagocytosis of stressed photoreceptors [14]. Additionally, TREM2-dependent activation of microglia promotes cisplatin-induced peripheral neuropathy in mice, and functional blockade of microglial TREM2 proves beneficial in mitigating this condition [15]. However, the role of TREM2 in the myelination and axonal growth of peripheral nerves has not been determined.

Schwann cells (SCs) are a type of glial cell in the PNS that exhibit similar neuroprotective functions to microglia, including immune response, phagocytosis, myelination, providing trophic support for neurons, and modulating synaptic pruning and plasticity [16–18]. SCs maintain the structure and function of peripheral nerves by ensheathment of unmyelinated axons, myelination of myelinated axons and secretion of neurotrophic factors [19]. Dysfunction initiates demyelination, axonal degeneration, and immune responses and causes peripheral neuropathy [20]. Considering the varying conditions and mechanisms found in peripheral neuropathy, the contribution of TREM2 to peripheral neuropathy may be multifaceted. Thus far, it is unclear how TREM2 regulates the function of SCs and influences peripheral nerve health.

In this study, we investigated the functional effects of TREM2 deficiency on SCs. Our results revealed that TREM2 is expressed in SCs but not in neurons in the PNS and that TREM2 deficiency impairs SC proliferation by impairing SYK and downstream signaling. Importantly, TREM2 deficiency leads to disruption of glycolytic flux and oxidative metabolism in SCs. The energy crisis caused by TREM2 deficiency induces mitochondrial damage and autophagy by activating AMPK and impairing PI3K-AKT-mTOR signaling. Furthermore, TREM2 deficiency exacerbates nerve lesions in a mouse model of acute motor axonal neuropathy (AMAN), a subtype of Guillain-Barré syndrome. TREM2 defects impair energy metabolism and axonal growth in the sciatic nerve, resulting in exacerbated neurological deficits and impaired nerve regeneration. Our findings demonstrated the critical role of TREM2 in the energy metabolism of SCs and in supporting nerve regeneration, indicating that TREM2 is a potential therapeutic target for peripheral neuropathy.

## Materials and methods

### Cell culture

Primary SCs were isolated from the sciatic nerves of mice aged 3–5 days as previously described [21]. Briefly, sciatic nerves were dissected, and the epineurium was removed. The tissue was then subjected to digestion with 0.05% collagenase/dispase (Roche, Mannheim, Germany) for 1 h and 0.25% trypsin (Gibco, NY, USA) for 15 min in a 37 °C, 5% CO_2_ incubator. Dissociated cells were cultured in culture medium containing DMEM/F12 medium supplemented with 10% FBS, 2 μM forskolin (Sigma, MA, USA), 10 ng/mL heregulin-β-1 (PeproTech, NJ, USA) and 1% penicillin/streptomycin (Gibco, NY, USA). After 48 h of cultivation, approximately half of the medium was replaced with fresh medium containing an additional 10 μM cytosine arabinoside (MCE, NJ, USA) for 2 days. Subsequently, the media was switched to culture medium for 3 days for further cell growth. Cells passage was digested with 0.125% trypsin. Cultured SCs exhibiting a purity exceeding 95%, as confirmed by immunostaining for S100β and nerve growth factor receptor (NGFR), were eligible for subsequent experiments (Figure S1).

Primary neurons were isolated from the spinal cords of embryonic 13.5-day-old fetal mice. Briefly, the spinal cord was initially dissected before the epineurium was removed. The tissue was then chopped into small slices and digested with tissue dissociation mix (Singleron, Nanjing, China) in a shaker at 37 °C for 15 min. After digestion, the cells were filtered with a 70 μm sterile filter and then centrifuged at 350 × *g* for 5 min. The cells were suspended in precooled PBS, and debris removal solution (Singleron, Nanjing, China) was added according to the manufacturer’s instructions. The mixture was further centrifuged at 450 × *g* for 10 min at 4 °C. The cells were suspended and washed with PBS. Finally, the cells were seeded in 12-well plates precoated with poly-L-lysine and cultured with NABG medium containing 97% neurobasal plus medium (Gibco, NY, USA), 2% B-27 Plus supplement (Gibco, NY, USA), 0.5% glutaMAX additive (Gibco, NY, USA), and 0.5% penicillin/streptomycin in a 37 °C, 5% CO_2_ incubator. Half of the culture medium was replaced with fresh NABG medium every two days. The cultured neurons were confirmed for subsequent experiments by using antibody immunostaining against TuJ1. The study was approved by the Ethics Committee of Medical Science Research of the Affiliated Hospital of Jining Medical University (NO. 2022B005).

### Cell transfection

Two lentiviral constructs with specific shRNAs were used to knockdown TREM2 (Obio Technology, Shanghai, China). The sequences of the TREM2 shRNA used were as follows: shTREM2-1: 5′-GTCATGTACTTATGACGCCTT-3′; shTREM2-2: 5′-GACCCTCTAGATGACCAAGAT-3′. A control shRNA served as the negative control. After 48 h of transfection, puromycin (2 μg/mL) was used to select for stable expression.

### Quantitative real-time PCR (qRT–PCR)

Total RNA was isolated from cultured SCs using TRIzol reagent (Invitrogen, CA, USA). Reverse transcription was performed using the PrimeScript™ II 1st Strand cDNA Synthesis Kit (Takara, Beijing, China). qRT‒PCR was conducted on an ABI QuantStudio5 system using UltraSYBR Mixture (CWBIO, Taizhou, China). The relative expression of the target gene was calculated using the 2^−ΔΔCt^ comparative quantification method, according to the Ct values of the target gene. Glyceraldehyde-3-phosphate dehydrogenase (GAPDH) was utilized as the normalization control. The sequences of the primers are listed in Supplementary Table 1.

### Cell Counting Kit-8 (CCK-8) assays

Cells were seeded into 96-well plates at a density of 2500 cells per plate. CCK-8 reagent was added to each well according to the manufacturer’s instructions (Dojindo, MD, USA). After incubation for 2–4 h at 37 °C, the absorbance at 450 nm was measured for each sample well using a microplate reader (BioTek Cytation Hybrid, Agilent, CA, USA).

### Western blotting

Total proteins were extracted from cultured SCs or sciatic nerves using RIPA lysis buffer (Beyotime, Beijing, China) supplemented with 1 mM PMSF (Solarbio, Beijing, China), 1 × phosphatase inhibitors (Solarbio, Beijing, China) and 1 × protease inhibitor cocktail (MCE, NJ, USA) on ice. After centrifugation and quantification, protein samples were separated and transferred onto PVDF membranes. After blocking using 5% skim milk for 1 h at room temperature, the membrane was incubated with primary antibodies overnight at 4 °C and then with HRP-conjugated secondary antibodies for 1 h at room temperature. Signals were detected by chemiluminescence solution (Proteintech, Wuhan, China) and quantified by ImageJ software (Version 1.4.9). Details of the primary and secondary antibodies are listed in Supplemental Table 2.

### Immunostaining

For cellular immunofluorescence, cells were fixed in 4% paraformaldehyde in PBS for 10 min and then permeabilized and blocked in 10% goat serum and 0.3% Triton X-100 in PBS at room temperature for 30 min. Subsequently, the cells were incubated with primary antibodies overnight at 4 °C, followed by incubation with Alexa Fluor 488-conjugated secondary antibodies and iFluor™ 647-phalloidin (Yeasen Biotechnology, Shanghai, China) for 2 h at room temperature. Finally, the cells were mounted in Fluoroshield with DAPI (Abcam, MA, USA). At least 10 independent fields of approximately 50 cells per field were captured from each group, and this process was repeated independently four times.

For frozen sectioning, mice were transcardially perfused with 4% paraformaldehyde. Sciatic nerves were dissected and postfixed in 4% paraformaldehyde for 2 h and then dehydrated in 30% sucrose for over 1 day until the tissue sank to the bottom. The tissues were embedded in optimal cutting temperature compound (Sakura Finetek, Torrance, USA) and cut into 10 μm longitudinal slices. Sections were permeabilized and blocked in 10% goat serum and 0.3% Triton X-100 in PBS for 1 h at room temperature. Subsequently, tissues were incubated with primary antibody overnight at 4 °C, followed by incubation with Alexa Fluor-conjugated secondary antibody for 1.5 h at room temperature. Finally, the tissues were mounted in antifade mounting medium and covered with cover slips. Images were captured using an Axio Imager Z2 microscope (Zeiss, Jena, Germany). At least 6 independent fields of view were captured per group from six independent experiments. The immunofluorescence intensity was quantified by ImageJ software. Details of the primary and secondary antibodies are listed in Supplemental Table 2.

### mtDNA copy number assay

Relative levels of mitochondrial DNA (mtDNA) copy number were determined by qRT‒PCR according to previously reported methods [22]. Briefly, total DNA was isolated using a genomic DNA kit (Tiangen, Beijing, China) and used for qRT‒PCR analysis. Mitochondrially encoded subunit 2 of cytochrome c oxidase (COX2) was used to measure mtDNA copy number and further normalized to the nuclear ribosomal protein s18. The sequences of the primers are listed in Supplementary Table 1.

### Energy metabolism analysis

A Seahorse XFe24 analyzer (Agilent Technologies, CA, USA) was applied to determine the extracellular acidification rate (ECAR) and oxygen consumption rate (OCR). For cells, cultured SCs were seeded in poly-L-lysine-coated Seahorse XFe24 plates at a density of 25,000 cells per well and incubated overnight. For tissues, sciatic nerves were rapidly dissected, and the epineurium was removed from anesthetized mice and then cut into small pieces. The tissues were dissociated with StemPro^TM^ accutase (Gibco, NY, USA) containing 0.1% DNase I (Roche, Mannheim, Germany) using a gentleMACS Dissociator (Miltenyi Biotec, Bergisch Gladbach, Germany), followed by filtration with a 70 μm sterile filter. Cells were seeded in poly-L-lysine-coated Seahorse XFe24 plates at 50,000 cells per well and centrifuged at 200 × *g* for 2 min. ECAR was measured under basal conditions and in response to 10 mM glucose, 1.5 μM oligomycin, and 50 mM 2-deoxyglucose to determine glycolysis, glycolytic capacity and glycolytic reserve. OCR was measured under basal conditions and in response to 1.5 μM oligomycin, 2.0 μM FCCP and 0.5 μM rotenone/antimycin A to determine mitochondrial basal respiration, ATP production, maximal respiration and spare capacity. Three measurements were conducted after the addition of each compound with 4 min mixing intervals, followed by 3 min measuring periods. Collected data were analyzed using Wave Desktop 2.6 software (Agilent Technologies) and normalized to total cell count or total protein content.

### Mitochondrial membrane potential (MMP) measurement

To determine the mitochondrial membrane potential, a JC-1 probe was applied following the manufacturer’s instructions (Abcam, MA, USA). Briefly, cultured SCs were washed with PBS and incubated with JC-1 working solution in the dark for 20 min at 37 °C. After washing twice with dilution buffer, fresh culture medium was added to the cells, which were then imaged by an inverted microscope Axio Observer 7 (Zeiss, Jena, Germany). The emission wavelengths of 590 nm and 529 nm were used to detect JC-1 aggregates and monomers, respectively. The fluorescence intensity of at least 10 visual fields was quantified using ImageJ software. The ratio of JC-1 aggregates to monomers was applied to quantify changes in the MMP.

### Transmission electron microscopy (TEM)

Cultured SCs were fixed with phosphate buffer containing 2.5% glutaraldehyde and 4% paraformaldehyde for 6 h and then postfixed in 1% aqueous osmium tetroxide for 1 h. Samples were dehydrated in increasing concentrations of alcohol and embedded in epoxy resin (SPI, NY, USA). Ultrathin sections (70 nm) prepared from the embedded tissues using an ultramicrotome (RMC-PT-PC, Tucson, AZ, USA) were stained with 2% uranyl acetate and 1% lead citrate. The ultrastructure was observed using a Tolas L120C electron microscope (Thermo Scientific, Waltham, MA, USA).

At least 10 independent fields at different layers of approximately 50 mitochondria per field were captured from each group, and this process was repeated independently six times. Mitochondrial length was defined as the longest longitudinal axis of the mitochondria. The length and average area of mitochondria were determined using ImageJ software.

### Measurement of glucose, glucose-6-phosphate, pyruvate and lactate

The fluorescent glucose analog 2-[N-(7-nitrobenz-2-oxa-1,3- diazol-4-yl)amino]-2-deoxy-D-glucose (2-NBDG) was applied to measure glucose uptake (Abcam, MA, USA). Cells were washed twice with PBS and incubated in glucose-free culture medium for 1 h at 37 °C. Subsequently, 100 μM 2-NBDG was added and incubated in the dark for 1 h. After incubation, the cells were collected and suspended in cell-based assay buffer. The levels of 2-NBDG uptake were determined by flow cytometry (Beckman, DxFLEX, CA, USA).

The levels of glucose-6-phosphate, lactate and pyruvate were measured using a high-sensitivity glucose-6-phosphate assay kit, a pyruvate assay kit and a lactate assay kit (all from Abcam, MA, USA), respectively, according to the manufacturer’s instructions. The absorbance was determined using a microplate reader at 450 nm for glucose-6-phosphate and at 570 nm for lactate and pyruvate.

### Intracellular ATP measurement

Intracellular ATP levels were measured in cell lysates using a Luminescent ATP Detection Assay Kit according to the manufacturer’s instructions (Abcam, MA, USA). Briefly, cultured SCs were added to detergent solution and incubated for 5 min in an orbital shaker at 600–700 rpm to lyse the cells and stabilize ATP. Subsequently, substrate solution was added and incubated for 5 min in an orbital shaker at 600–700 rpm. The mixture was further incubated for 10 min in the dark and measured by a microplate reader at 570 nm.

### Nicotinamide adenine dinucleotide (NAD+) measurement

The levels of NAD^+^ and its reduced form NADH were measured using a WST-8-based NAD^+^/NADH assay kit (Beyotime, Beijing, China). Briefly, cultured SCs were washed twice with PBS and lysed using NAD^+^/NADH extraction buffer. Samples were centrifuged at 12,000 × *g* for 10 min, and the supernatant was collected. For the measurement of total NAD^+^ and NADH concentrations, working solution was added to the cell lysis supernatant and incubated in the dark at 37 °C for 10 min. Subsequently, color developing agent was added to the plate for further incubation at 37 °C for 30 min. The absorbance was measured at 450 nm using a microplate reader. For the measurement of NADH, the supernatant was incubated at 60 °C for 30 min, and then the above procedure was performed. The levels of NAD^+^ were derived by subtracting NADH from total NAD^+^ and NADH.

### Mitochondrial reactive oxygen species (ROS) measurement

The levels of mitochondrial ROS were determined by immunostaining and flow cytometry. For immunofluorescence staining, cells were washed twice with PBS and incubated with 2 μM MitoSOX Red (MCE, CA, USA) and 100 nM MitoTracker Green (Thermo Fisher, MA, USA) in the dark for 30 min. The stained cells were then washed with PBS and observed using an inverted microscope Axio Observer 7. The fluorescence intensity of at least 10 visual fields per group was quantified using ImageJ software. For flow cytometry analysis, cells were collected and incubated with 2 μM MitoSOX Red in the dark for 30 min. The stained cells were then washed twice with PBS and analyzed by flow cytometry.

### Lysosome measurement

The levels of lysosomes were determined by immunostaining and flow cytometry. For immunofluorescence staining, cells were washed twice with PBS and incubated with 1 μM LysoTracker Green and 100 nM MitoTracker Red (all from Beyotime Biotechnology, Beijing, China) in the dark for 30 min. The stained cells were then washed with PBS and observed by an inverted microscope Axio Observer 7. The fluorescence intensity of at least 10 visual fields per group was quantified using ImageJ software. For flow cytometry analysis, cells were collected and incubated with 2 μM LysoTracker Green in the dark for 30 min. The stained cells were then washed twice with PBS and analyzed by flow cytometry.

### Metabolomics

Targeted energy metabolomics was performed by MetWare Biotechnology (Wuhan, China) using liquid chromatography-tandem mass spectrometry. Briefly, 2 × 10^6^ cultured SCs were collected and mixed with 500 µL of 80% methanol/water (precooled at −20 °C), followed by vortexing at 2500 rpm/min for 2 min. The samples were frozen in liquid nitrogen for 5 min and incubated on ice for 5 minu and vortexed for another 2 min. After three repetitions, the samples were centrifuged at 12,000 × *g* for 10 min at 4 °C, and the supernatants were collected and incubated at -20 °C for 30 min. After anther centrifugation at 12,000 × *g* for 10 min at 4 °C, the supernatant was subjected to mass spectrometry analysis with a QTRAP 6500 mass spectrometer (AB-SCIEX, MA, USA). The peak area and retention time were extracted using Multiquanta software and normalized into standard energy metabolizers for metabolite identification. Significantly regulated metabolites between groups were identified by fold change (≥2 or ≤0.5) and variable importance of projection (≥1). The Kyoto Encyclopedia of Genes and Genomes (KEGG) database was used to analyze enriched pathways.

### GD1a-IgG purification

Plasma was collected from a patient diagnosed with GD1a-IgG seropositive Guillain‒Barré syndrome. Plasma was filtered to remove impurities and dialyzed into PBS. IgG was purified using the ÄKTA Pure Protein Purification System (Cytiva, Washington, USA) with a HiTrapTM Protein G HP chromatography column (GE Healthcare, IL, USA). The purified IgG was dialyzed into PBS and adjusted to a concentration of 10 mg/mL.

### AMAN animal model and treatment

GD3s^-/-^ mice that overexpress GD1a were generated using the traditional embryonic stem cell shooting technique on the C57BL/6 J background by Cyagen Biosciences (Guangzhou, China) [23]. Mice were maintained under a 12-h light/dark cycle at a controlled temperature and were fed and watered freely. The AMAN animal model was produced as described previously [24]. The right sciatic nerves of GD3s^-/-^ mice aged 8–12 weeks were crushed 35 mm to the middle toe for 30 s with fine forceps with carbon on day 0. Intraperitoneal injections of GD1a-IgG (total 2 mg per mouse) were administered on days 3, 5, 7, 9, 11, and 13 after surgery. Behavioral and electrophysiological tests and nerve collection were performed 21 days after surgery.

To decrease the expression of TREM2, siRNA injected in situ using Entranster^TM^-in vivo following the manufacturer’s instructions (Engreen, Beijing, China). The sequence of TREM2 siRNA (siTREM2) was as follows: 5′-GTCATGTACTTATGACGCCTT-3′. A negative control siRNA (siNC) was used as a control. A mixture of 2.5 μL of siRNA (5 μg) and 2.5 μL of Entranster^TM^ was prepared, kept at room temperature for 15 min, and then locally injected into the right sciatic nerves of mice 3 days prior to surgery.

### Electrophysiology

Mice were anesthetized using 2–3% isoflurane, and the sciatic nerve was exposed for electrophysiological testing. The stimulating electrode was positioned at the sciatic nerve trunk, the recording electrode was positioned at the abdomen of the gastrocnemius muscle, the reference electrode was positioned at the Achilles tendon, and the ground electrode was placed in the tail. The nerve was stimulated at least three times with a pulse duration of 0.1 ms and a frequency of 1 Hz to record the amplitude and latency of the complex muscle action potential (CMAP).

### Catwalk gait

A CatWalk automated gait analysis system was applied to assess the motor function of mice (Zhongshi Dichuang Technology, Beijing, China). Mice underwent adaptive training and dieting prior to measurement. High-speed digital cameras were employed to record the footsteps as the mice crossed the runway. The collected data encompassed various parameters that evaluate animal balance, coordination and strength, including stand, max contact area, max intensity, mean intensity, swing, swing speed, stride length, step cycle, paw angle body axis, body speed and paw drag. The sciatic nerve function index (SFI) was measured noninvasively to monitor the recovery of the sciatic nerve after injury. SFI was calculated using the following formula [25]: SFI = 118.9 (ETS - NTS)/NTS - 51.2 (EPL - NPL) NPL - 7.5. Toe spread (TS), the distance between the first and fifth toes; print length (PL), the distance between the third toe and the heel; E, the experimental side; N, the normal side. A lower SFI indicates more severe functional impairment of the sciatic nerve.

### Statistical analysis

Data are presented as the mean ± standard deviation. Statistical analysis was carried out using GraphPad Prism 9. The Shapiro‒Wilk test was used for normality analysis of the data distribution. Two or more groups of data were compared by two-tailed Student’s *t* test or one-way analysis of variance ANOVA followed by Bonferroni posttest. *P* < 0.05 was considered statistically significant.

## Results

### TREM2 is localized in SCs and its deficiency impairs SC proliferation

**Fig. 1:**
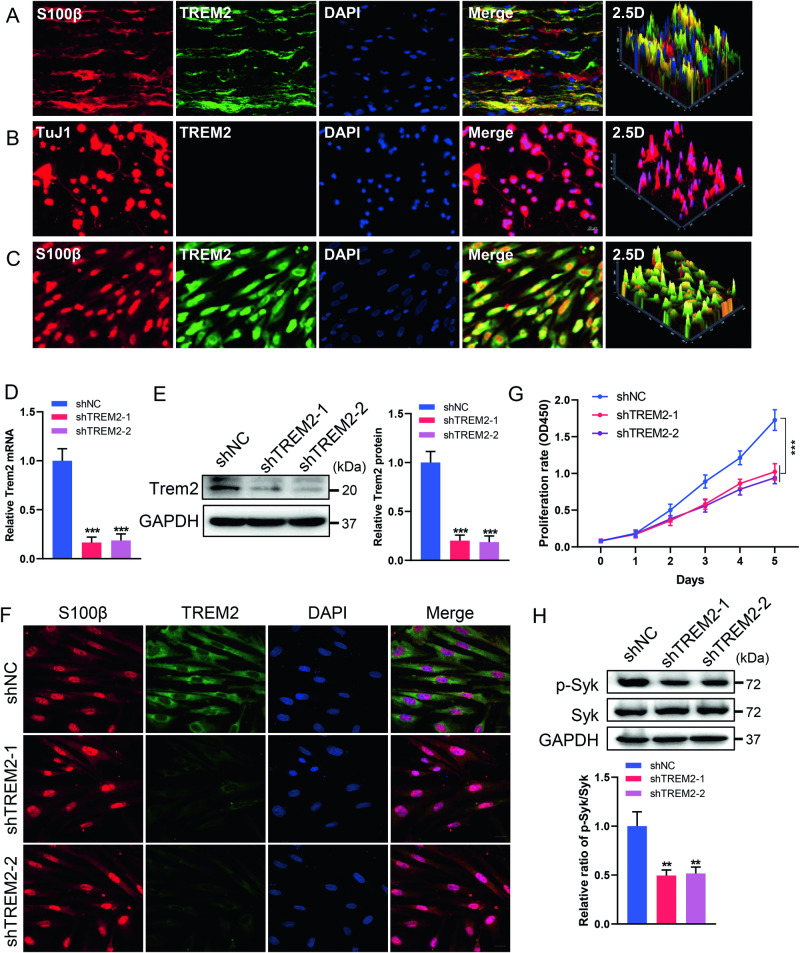
TREM2 deficiency impairs SC proliferation. **A**–**C** Immunostaining identified the localization of TREM2 in the sciatic nerve, primary neurons and primary SCs from C57BL/6 J mice. S100β- and TuJ1-positive immunostaining indicate SCs and neurons, respectively. Scale bar, 20 μm. **D**–**F** The expression of TREM2 was knocked down by transfecting TREM2-specific shRNAs into primary SCs. Identification of the expression of TREM2 by qRT‒PCR (**D**), western blot (**E**) and immunofluorescence staining (**F**). Scale bar, 20 μm. *n* = 3. **G** Cell proliferation was determined by the Cell Counting Kit-8 assay. *n* = 4. **H**. Levels of phosphorylated (Tyr525/526) and total SYK in SCs were determined by western blot. *n* = 3. **p < 0.01, ***p < 0.001.

TREM2 has been reported to be predominantly expressed on microglia in the CNS [1]. However, its expression in PNS remains unclear. We first investigated the expression of TREM2 in the sciatic nerve by immunofluorescence staining. S100β was used as a marker for SCs. The results demonstrated that TREM2 was primarily expressed on SCs (Fig. 1A). Furthermore, primary neurons and SCs were isolated to identify the localization of TREM2. TUJ1 was used as a marker for neurons. The results verified that TREM2 was expressed in SCs but not in neurons (Fig. 1B, C).

To further confirm the role of TREM2 in mediating the functions of SCs, we employed two specific shRNAs to downregulate the expression of TREM2 in primary SCs. The efficiency of TREM2 knockdown was verified by qRT‒PCR, western blot and immunofluorescence staining (Fig. 1D–F). To assess the influence of TREM2 deficiency on SC proliferation, a CCK-8 assay was performed. Compared with the control group, TREM2 knockdown significantly impaired cell proliferation (Fig. 1G).

TREM2/DAP12 activation recruits SYK kinases to trigger downstream signal transduction, notably activating the PI3K signaling pathway, mediating autophagy and maintaining the metabolic balance of glial cells [4, 26]. Our findings showed that TREM2 knockdown significantly reduced the phosphorylation of SYK (Fig. 1H). The downregulation of SYK kinase activity may imply disruption of related biological processes in SCs, impairing cell proliferation.

### TREM2 deficiency altered energy-related metabolic pathways

**Fig. 2:**
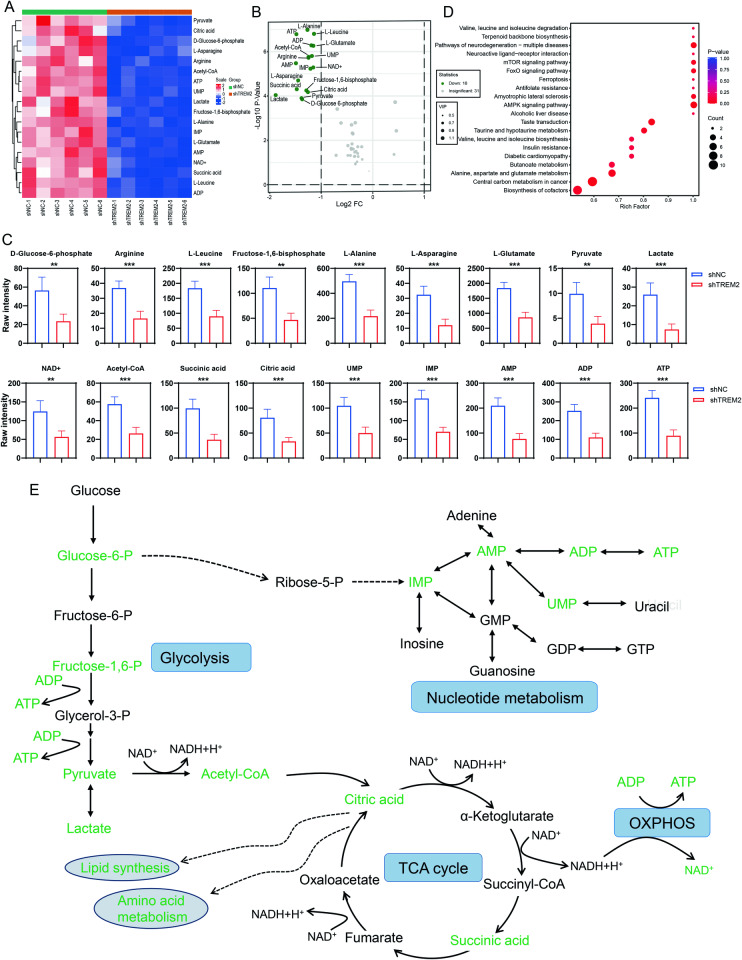
Detailed effects of TREM2 deficiency on the energy metabolism of SCs. **A** Heatmap of differentially abundant metabolites induced by TREM2 deficiency in SCs according to energy metabolomics analysis. **B** Volcano map of the differentially abundant metabolites. **C** Quantification of the differentially abundant metabolites. **D** Kyoto Encyclopedia of Genes and Genomes (KEGG) enrichment pathway analysis based on the differentially abundant metabolites. **E** Summary of the metabolic pathways and targets affected by TREM2 deficiency in SCs. A schematic representation of the metabiotic pathways of glycolysis, the tricarboxylic acid (TCA) cycle, oxidative phosphorylation (OXPHOS) and purine/pyrimidine metabolism is shown. There were significantly fewer metabolites in TREM2-deficient SCs than in control SCs, as indicated in green. Glucose-6-P glucose-6-phosphate, Fructose-6-P fructose-6-phosphate, Fructose-1,6-P fructose-1,6-bisphosphatase, Glycerol-3-P glycerol-3-phosphate, DAD^+^ nicotinamide adenine dinucleotide, Acetyl-CoA acetoacetyl coenzyme A, GMP guanosine-5′-monophosphate, GDP guanosine-5′-monophosphate, GTP guanosine-5′-monophosphate, UMP uridine monophosphate, IMP inosine monophosphate, AMP adenosine monophosphate, ADP adenosine diphosphate, ATP adenosine triphosphate. **p < 0.01, ***p < 0.001.

Energy metabolism plays an indispensable role in SC proliferation, supporting axonal growth and myelination. Targeted energy metabolomics analysis was first performed to evaluate the effects of TREM2 deficiency on the energy-related metabolic pathways of SCs. A total of 50 metabolites were identified and subjected to cluster analysis, principal component analysis (PCA) and partial least squares-discriminant analysis (OPLS-DA) (Fig. S2A-C). The heatmap clustering and the score plots of PCA and OPLS-DA showed discernible separation between the sample groups, indicating significant differences in the metabolome across the groups. The cluster heatmap and volcano plot depicting the significantly altered metabolites in the energy metabolism pathway are shown in Fig. 2A, B. Specifically, TREM2 knockdown significantly decreased the levels of glycolytic metabolites (glucose 6-phosphate, fructose-1,6-bisphosphate, pyruvate and lactate), tricarboxylic acid (TCA) cycle metabolites (acetyl-CoA, citric acid and succinate) and oxidative phosphorylation (OXPHOS) metabolites (ATP and NAD^+^) (Fig. 2C). In addition, TREM2 knockdown also resulted in decreased levels of amino acids critical for protein synthesis (arginine, L-leucine, L-alanine, L-asparagine, and L-glutamate), as well as a decline in the abundance of purine and pyrimidine metabolites critical for nucleotide synthesis and energy supply (IMP, UMP, AMP, and ADP) (Fig. 2C). Correlation analysis indicated close metabolic proximity and synergistic interactions among these altered metabolites (Figure S2,D). KEGG pathway enrichment analysis showed that TREM2 deficiency was involved in the AMPK signaling pathway, mTOR signaling pathway, forkhead box O (FoxO) signaling pathway, neuroactive ligand‒receptor interaction, pathways of neurodegeneration, valine, leucine and isoleucine degradation, terpenoid backbone biosynthesis, and alanine, aspartate and glutamate metabolism (Fig. 2D). Further regulatory interaction network analysis demonstrated that TREM2 deficiency was associated with the TCA cycle, pentose phosphate pathway, HIF-1 signaling pathway, PI3K-AKT signaling pathway, DNA replication and RNA degradation (Figure S3). By enriching these altered metabolites in energy-related metabolic pathways, it can be inferred that TREM2 deficiency impairs glycolysis, the TCA cycle, OXPHOS, nucleotide metabolism, amino acid metabolism and lipid synthesis in SCs (Fig. 2E).

### TREM2 deficiency impairs the glycolytic flux of SCs

**Fig. 3:**
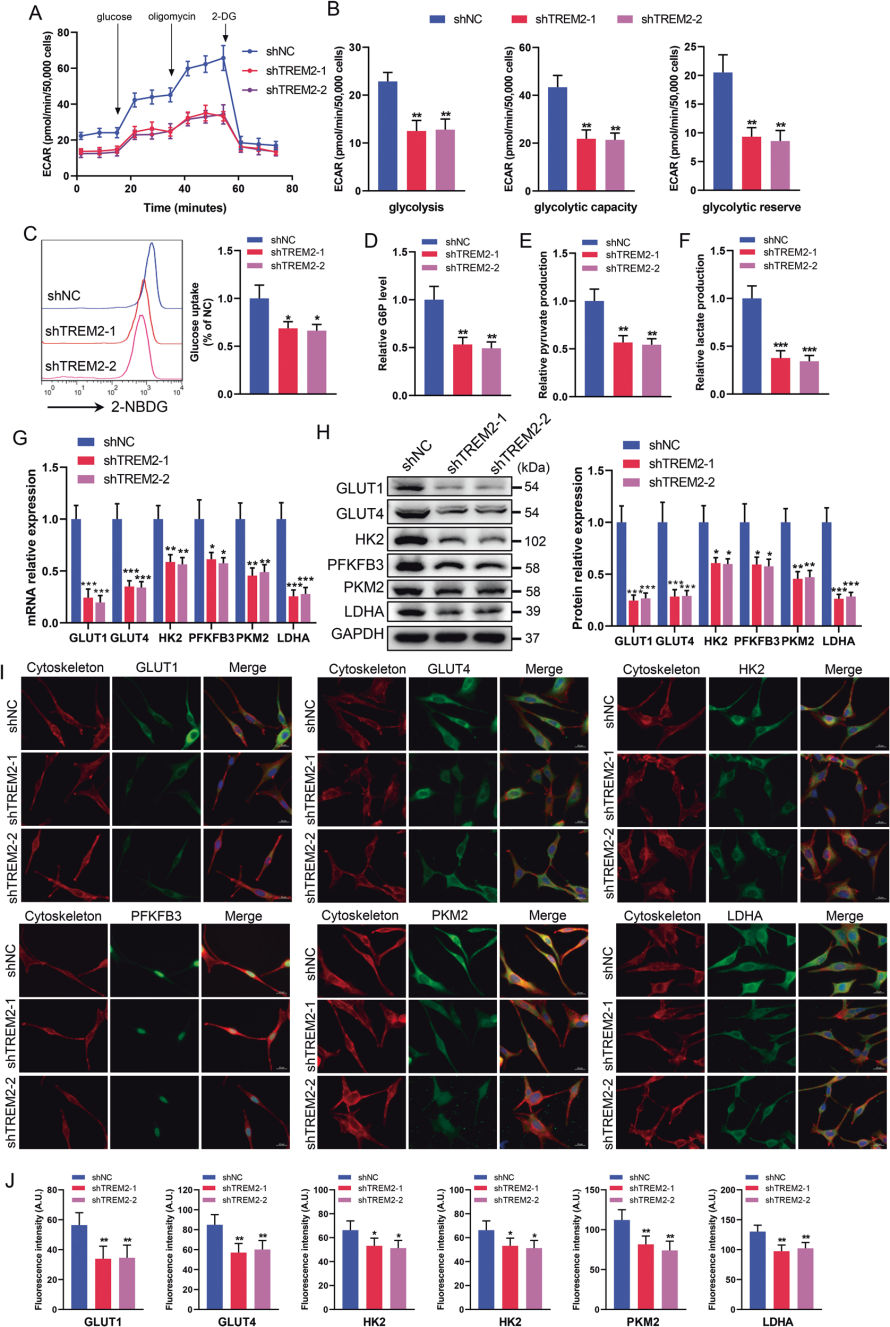
TREM2 deficiency impairs SC glycolytic flux. **A** The extracellular acidification rate (ECAR) of SCs was determined by Seahorse experiments. **B** Quantitative analysis of glycolysis, glycolytic capacity and glycolytic reserve in ECAR. *n* = 3. **C** Glucose uptake was measured by flow cytometry using 2-[N-(7-nitrobenz-2-oxa-1,3- diazol-4-yl)amino]-2-deoxy-D-glucose (2-NBDG). *n* = 4. Measurement of glycolytic products of glucose-6-phosphate (G6P) (**D**), pyruvate (**E**) and lactate (**F**) in SCs. *n* = 4. qRT‒PCR (**G**) and western blot (**H**) analysis of the expression levels of glycolysis-related genes in SCs, including glucose transporter 1/4 (GLUT)1/4, hexokinase 2 (HK2), 6-Phosphofructo-2-kinase/fructose-2,6-bisphosphatase 3 (PFKFB3), Pyruvate kinase 2 (PKM2) and lactate dehydrogenase A (LDHA). *n* = 4. **I**, **J** Representative immunofluorescence and quantification of glycolysis-related proteins in SCs. Scale bar, 20 μm. *N* = 4, *n* ≥ 10 fields/group. *p < 0.05, **p < 0.01, ***p < 0.001.

We conducted further investigation into the detailed effects of TREM2 deficiency on the glycolytic flux of SCs. For real-time analysis of glycolysis, ECAR was conducted using a Seahorse XFe24 analyzer. The results showed that TREM2 knockdown resulted in a marked decrease in glycolysis, glycolytic capacity and reserve compared to the control group (Fig. 3A, B). Additionally, we measured the levels of glucose uptake and glycolytic metabolites, showing that TREM2 knockdown significantly reduced glucose uptake as well as the levels of intracellular glucose-6-phosphate, pyruvate and lactate (Fig. 3C–F).

We subsequently evaluated the impact of TREM2 deficiency on the expression of glycolysis-related genes in SCs. Glucose uptake depends on a family of glucose transporters (GLUTs) [27]. Following uptake, glucose is phosphorylated to glucose-6-phosphate by hexokinase (HK). 6-Phosphofructo-2-kinase/fructose-2,6-bisphosphatase 3 (PFKFB3), a key glycolytic regulator enzyme and a known “regulator” of human metabolism, catalyzes the conversion of fructose-6-phosphate to fructose-2,6-bisphosphate, an allosteric activator of phosphofructokinase-1 [28]. Pyruvate kinase (PKM), a rate-limiting enzyme, catalyzes the conversion of phosphoenolpyruvate to pyruvate [29]. The conversion of pyruvate to lactate is facilitated by lactate dehydrogenase A (LDHA) [30]. Our findings indicated that TREM2 knockdown significantly reduced the mRNA and protein levels of GLUT1 and GLUT4 (Fig. 3G–J), potentially contributing to decreased glucose intake. Moreover, the mRNA and protein levels of HK2, PFKFB3, PKM2 and LDHA were markedly lower in the TREM2 knockdown groups than in the control group (Fig. 3G–J), which may explain the decreased glycolytic products. Altogether, these results suggest that TREM2 deficiency impairs the glycolytic flux of SCs by decreasing glucose uptake and the levels of glycolytic enzymes.

### TREM2 deficiency impairs the oxidative metabolism of SCs

**Fig. 4:**
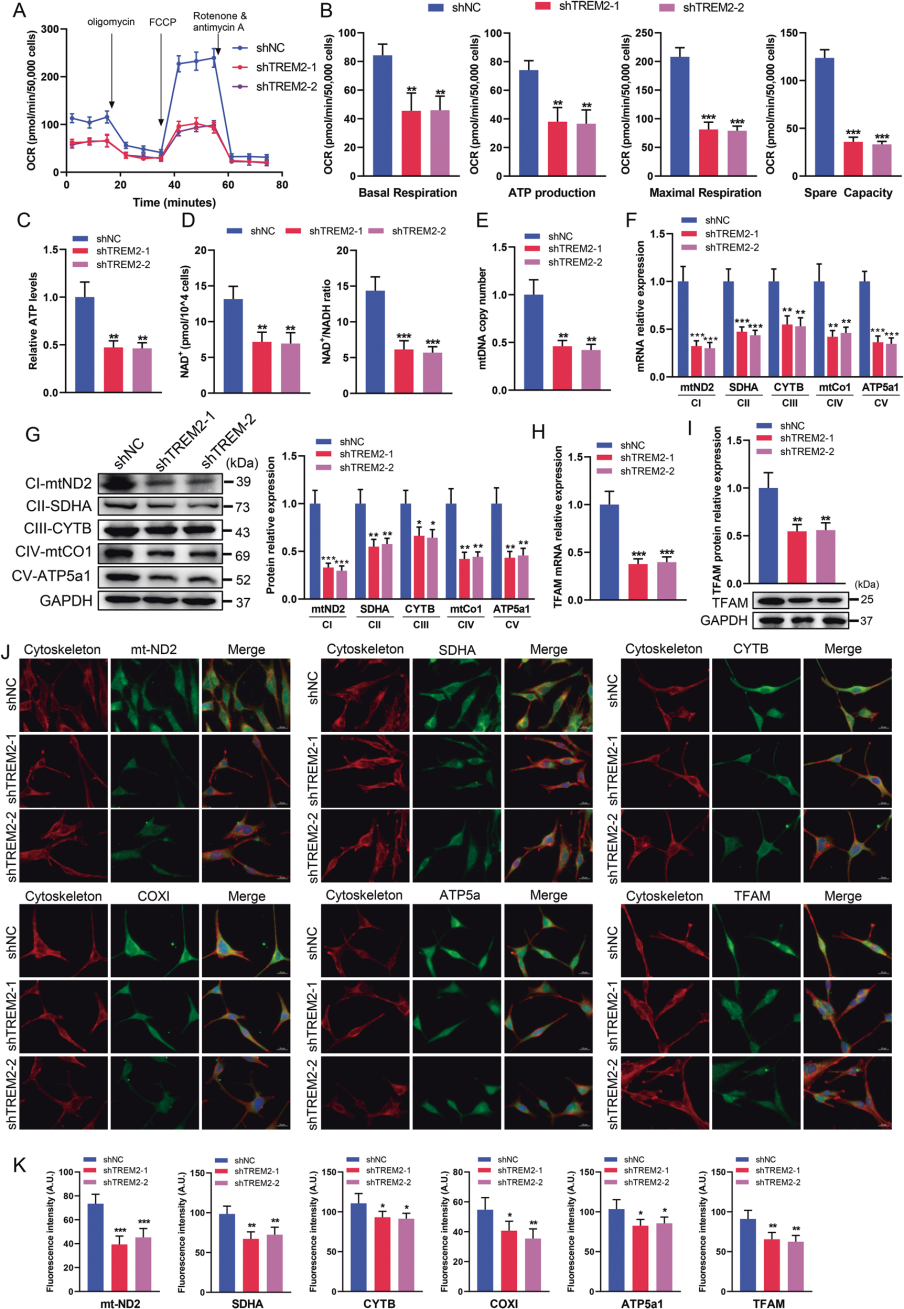
TREM2 deficiency impairs oxidative metabolism in SCs. **A** The oxygen consumption rate (OCR) of SCs was determined by Seahorse experiments. **B** Quantitative analysis of basal respiration, ATP production, maximal respiration, and spare respiratory capacity in OCR. *n* = 3. **C** Measurement of ATP production by chemiluminescence. *n* = 4. **D** The levels of nicotinamide adenine dinucleotide (NAD^+^) and its ratio to NADH in SCs were determined by chemiluminescence. *n* = 4. **E** Measurement of mitochondrial DNA (mtDNA) copy number in SCs by qRT‒PCR. *n* = 3. qRT‒PCR (**F**) and western blot (**G**) analysis of the expression levels of mitochondrial electron transport chain (ETC) components in SCs, including mitochondrially encoded NADH dehydrogenase 2 (mt-ND2), succinate dehydrogenase complex flavoprotein subunit A (SDHA), mitochondrially encoded cytochrome b (mt-CYTB), mitochondrially encoded cytochrome C oxidase I (mt-CO1), and mitochondrially encoded ATP synthase alpha-subunit (mt-ATP5a1). CI, CII, CIII, CIV and CV represent mitochondrial ETC complexes I, II, III, IV and V, respectively. *n* = 3. qRT‒PCR (**H**) and western blot (**I**) analysis of the expression levels of transcription factor A (TFAM) in SCs. *n* = 3. **J**, **K** Representative immunofluorescence and quantification of mitochondrial electron transfer chain proteins and TFAM in SCs. Scale bar, 20 μm. *N* = 4, n ≥ 10 fields/group. *p < 0.05, **p < 0.01, ***p < 0.001.

We conducted real-time analysis of mitochondrial respiration function by measuring the OCR. The results demonstrated a significant decrease in mitochondrial basal respiration, ATP production, maximal respiration and spare capacity in the TREM2 knockdown groups (Fig. 4A, B). The decreased intracellular ATP concentration caused by TREM2 deficits was further confirmed by chemiluminescence (Fig. 4C). NAD^+^ plays a crucial role as a carrier for electrons and hydrogen, as well as a coenzyme for redox reactions, and its level determines the energy metabolism state of cells [31, 32]. Therefore, we examined the intracellular levels of NAD^+^ and NADH, and found a significant reduction in the NAD^+^ level and the NAD^+^/NADH ratio in the TREM2 knockdown groups (Fig. 4D).

The mtDNA copy number serves as an indicator of mitochondrial mass and a biomarker for disease progression [33]. The function of mitochondrial respiration depends on respiratory chain complexes [34]. We examined both the mtDNA copy number and mRNA levels of mitochondrial electron transport chain (ETC) components, including mitochondrially encoded NADH dehydrogenase 2 (mt-ND2), succinate dehydrogenase complex flavoprotein subunit A (SDHA), mitochondrially encoded cytochrome b (mt-CYTB), mitochondrially encoded cytochrome C oxidase I (mt-CO1), and mitochondrially encoded ATP synthase alpha-subunit (mt-ATP5a1). The results showed that the mtDNA copy number and the mRNA levels of mt-ND2, SDHA, mt-CYTB, mt-CO1 and mt-ATP5a1 were significantly decreased in the TREM2 knockdown groups (Fig. 4E, F). Western blot and immunostaining further showed that TREM2 knockdown significantly reduced the protein levels of mt-ND2, SDHA, mt-CYTB, mt-CO1 and mt-ATP5a1 (Fig. 4G, J, K). In addition, we evaluated the mRNA and protein levels of mitochondrial transcription factor A (TFAM) and found a significant reduction in TFAM expression due to TREM2 knockdown (Fig. 4H–K), which may contribute to the decrease in mtDNA copy number and mitochondrial coding respiratory complex components. Altogether, these results indicate that TREM2 deficiency impairs the oxidative metabolism of SCs by reducing the expression levels of TFAM, mtDNA copy number and ETC components.

### TREM2 deficiency induced mitochondrial damage and autophagy

**Fig. 5:**
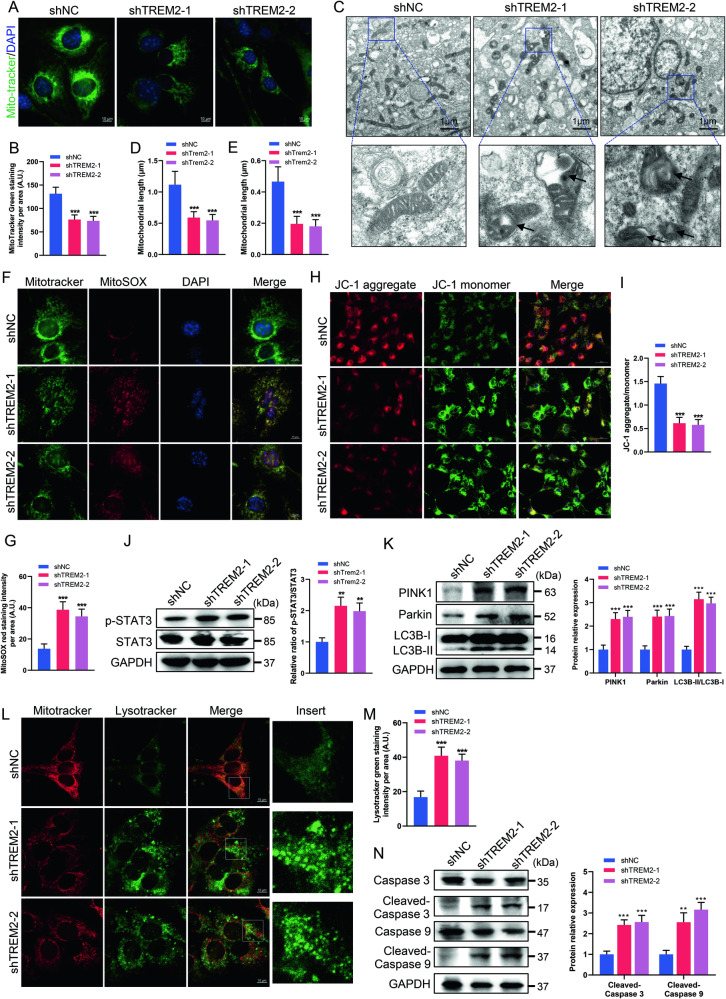
TREM2 deficiency induces mitochondrial damage and autophagy. **A** Representative fluorescence images of mitochondria in SCs stained with MitoTracker Green. Scale bars, 10 μm. **B** Quantification of mitochondrial intensity, indicating the number of mitochondria. *N* = 4, *n* ≥ 10 fields/group. **C**–**E** Representative electron microscopy images and quantification of mitochondrial length and area in SCs. Scale bars, 1 μm. *N* = 6, *n* ≥ 10 fields/group. **F** Mitochondria were stained with MitoTracker Green and MitoSOX Red to evaluate the production of mitochondrial reactive oxygen species. **G** Quantification of MitoSOX Red fluorescence. Scale bars, 10 μm. *N* = 4, *n* ≥ 10 fields/group. **H** The mitochondrial membrane potential (MMP) in SCs was measured by JC-1 staining as an indicator of mitochondrial activity. JC-1 aggregates indicate a high MMP, while JC-1 monomers indicate a low MMP. Scale bars, 50 μm. **I** Quantification of the ratio of JC-1 aggregates/monomers. *N* = 4, *n* ≥ 10 fields/group. **J** Levels of phosphorylated (Tyr705) and total signal transductors and transcriptional activator 3 (STAT3) in SCs were determined by western blot. *n* = 3. **K** The levels of PTEN-induced putative kinase 1 (PINK1) and parkin and the LC3BII/LC3BI ratio in SCs were determined by western blot. *n* = 3. **L** Mitochondria and lysosomes were stained with MitoTracker Red and LysoTracker Green, respectively. Scale bars, 10 μm. **M** Quantification of LysoTracker Green fluorescence. *N* = 4, *n* ≥ 10 fields/group. **N** Levels of cleaved-caspase 3, total caspase 3, cleaved-caspase 9 and total caspase 9 were determined by western blot. *n* = 3. **p < 0.01, ***p < 0.001.

The energy crisis caused by TREM2 deficiency prompted us to explore the consequences of TREM2 knockdown on mitochondrial mass. MitoTracker staining showed that the number of mitochondria was significantly decreased in the TREM2 knockdown groups (Fig. 5A, B). Additionally, TEM was employed to visualize mitochondrial structural features, which demonstrated that TREM2 knockdown markedly reduced both the length and area of mitochondria (Fig. 5C–E). Intriguingly, we observed the presence of mitochondrial lesions, such as swelling and fragmentation, compromised membrane integrity, and disrupted or absent cristae, in the TREM2 knockdown groups (Fig. 5C). Mitochondrial damage can induce the production of mitochondrial ROS [35]. Subsequently, we performed immunofluorescence staining and flow cytometry with red fluorescent MitoSOX dye to examine the effect of TREM2 knockdown on mitochondrial ROS production in SCs. Comparatively, TREM2 knockdown resulted in a significant increase in mitochondrial ROS levels compared to those in the control group (Fig. 5F, G and Fig. S4A). Furthermore, to further investigate the potential effects of TREM2 knockdown on mitochondrial activity, we assessed the mitochondrial membrane potential (MMP) using a JC-1 probe. The results revealed a significant decrease in the JC-1 aggregate/monomer ratio in the TREM2 knockdown groups, indicating a substantial reduction in the MMP (Fig. 5H, I).

Signal transductors and transcriptional activators 3 (STAT3) is thought to function as a direct transcription factor that regulates a multitude of genes associated with mitochondrial respiration, ROS production and autophagy [36, 37]. Here, we examined the expression and phosphorylation of STAT3 and found that TREM2 knockdown increased the phosphorylation (Tyr705) of STAT3 (Fig. 5J). Concurrently, activated STAT3 induced autophagy. When ROS causes mitochondrial damage, PTEN-induced putative kinase 1 (PINK1) stabilizes the formation of a large complex on the outer mitochondrial membrane, where it recruits parkin to the damaged mitochondria [38]. Parkin anchored to mitochondria is then further ubiquitinated to facilitate the recognition of impaired mitochondria, triggering mitophagy [39]. During autophagosome formation, the LC3-I protein is converted into its autophagic vesicle-associated form (LC3-II), serving as a vital marker of autophagic activity within cells [40]. Our results demonstrated that the levels of PINK1 and parkin and the LC3BII/LC3BI ratio were significantly increased in the TREM2 knockdown groups compared to the control group, indicating that TREM2 deficiency increased autophagy (Fig. 5K). Consistently, immunofluorescence analysis revealed an accumulation of autophagosomes positive for PINK1 and LC3B in the TREM2 knockdown groups (Fig. S4B, C). Moreover, the heightened presence of lysosomes, as confirmed by immunofluorescence staining and flow cytometry analysis, further supported the notion that TREM2 knockdown induces autophagy (Fig. 5L, M and Fig. S4D). Given that TREM2 deficiency impairs mitochondrial function and results in a decrease in the MMP, it is speculated that it also triggers mitochondria-dependent apoptosis. Caspases, a type of cysteine protease, are known to play a significant role in mediating mitochondria-dependent apoptosis, particularly caspase-3 and caspase-9 [41]. As expected, TREM2 knockdown increased the cleavage levels of caspase-3 and caspase-9, indicating the upregulation of apoptosis (Fig. 5N). Overall, our findings suggest that TREM2-deficient SCs exhibit defects in energy metabolism, along with increased mitochondrial damage and autophagy.

### TREM2 deficiency activates AMPK and impairs PI3K-AKT-mTOR signaling

**Fig. 6:**
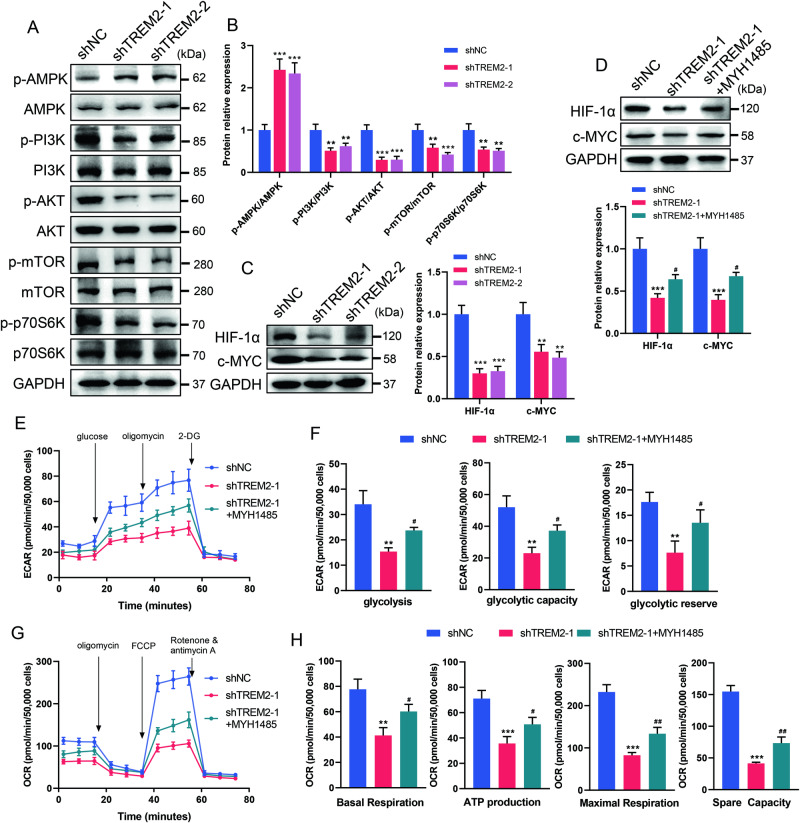
TREM2 deficiency activates AMPK and impairs PI3K-AKT-mTOR signaling. **A**, **B** Levels of phosphorylated (Thr172) and total AMPK, phosphorylated (Tyr607) and total PI3K, phosphorylated (Ser473) and total AKT, phosphorylated (Ser2448) and total mTOR, and phosphorylated (Thr389/Thr412) and total p70S6K were determined by western blot. *n* = 3. **C** Levels of HIF-1α and c-MYC were determined by western blot. *n* = 3. **D**–**H** TREM2-deficient SCs (shTREM2-1) were treated with 5 μM MHY1485, an mTOR activator. The expression of HIF-1α and c-MYC was determined by western blot (**D**). *n* = 3. Extracellular acidification rate (ECAR) and oxygen consumption rate (OCR) were determined by Seahorse experiments (**E**, **G**). Quantitative analysis of glycolysis, glycolytic capacity and glycolytic reserve in the ECAR (**F**) and basal respiration, ATP production, maximal respiration, and spare respiratory capacity in the OCR (**H**). *n* = 3. **p < 0.01, ***p < 0.001, compared with shNC; ^#^*p* < 0.05, ^##^*p* < 0.01, compared with shTREM2-1.

In the CNS, TREM2-DAP12 enhances the anabolism and energy production required for microglial activation by activating the PI3K-AKT-mTOR signaling pathway [42]. We therefore asked whether TREM2 deficiency causes energy impairment in SCs through a similar mechanism. First, we examined whether TREM2 deficiency activates AMPK and impairs the PI3K-AKT-mTOR pathway. The results showed that TREM2 knockdown was associated with increased phosphorylation of AMPK and reduced phosphorylation of PI3K, AKT and mTOR, which are implicated in p70S6K activation, protein synthesis and cell cycle progression (Fig. 6A, B). The impairment of PI3K-AKT-mTOR signaling is responsible for energy crisis, as well as the activation of mitochondrial autophagy, which is consistent with the findings of previous studies [4]. Regulation of glycolysis and OXPHOS gene expression is controlled by metabolic “master regulators” such as HIF-1α and c-MYC [43, 44]. Thus, it is worth investigating the effect of TREM2 deficiency on these regulators. The results showed that the cellular levels of HIF-1α and c-MYC were markedly reduced in the TREM2 knockdown groups, indicating suppressed transcription of glycolysis and OXPHOS genes (Fig. 6C).

To confirm the role of PI3K-AKT-mTOR signaling in energy metabolism and mitochondrial autophagy, we employed an mTOR signaling pathway activator, MYH1485, in a TREM2 knockdown group. Upon MYH1485 treatment, the expression of HIF-1α and c-MYC was restored in the TREM2 knockdown group (Fig. 6D). MYH1485 rescued the mRNA expression of GLUT1, GLUT4, HK2, PFKFB3, PKM2, and LDHA and restored the levels of glucose uptake and intracellular glucose-6-phosphate, pyruvate, and lactic acid production in the TREM2 knockdown group, suggesting that the activation of mTOR signaling reversed impaired glycolysis (Fig. S5A–E). Seahorse analysis of the ECAR showed that MYH185 rescued the impaired glycolysis, glycolytic capacity and glycolytic reserve of TREM2-deficient SCs (Fig. 6E, F). The mtDNA copy numbers and mRNA expression of TFAM and mitochondrial ETC complexes were rescued upon MYH1485 treatment in the TREM2 knockdown group, indicating a restored mitochondrial respiration (Fig. S5F–H). The results were confirmed by a rescued OCR in Seahorse examination, including partially restored basal respiration, ATP production, maximum respiration and respiratory capacity (Fig. 6G, H). Chemiluminescence analysis demonstrated that MYH1485 partially reversed the changes in the intracellular ATP concentration, NAD^+^ level and NAD^+^/NADH ratio in the TREM2 knockdown group (Fig. S5I–K). Moreover, MYH1485 restored the number of mitochondria and reversed the decreases in the length and area of mitochondria in the TREM2 knockdown group (Fig. S6A–D). These results indicate that TREM2 deficiency reduces anabolic and energy production by activating AMPK and impairing PI3K-AKT-mTOR signaling.

**Fig. 7:**
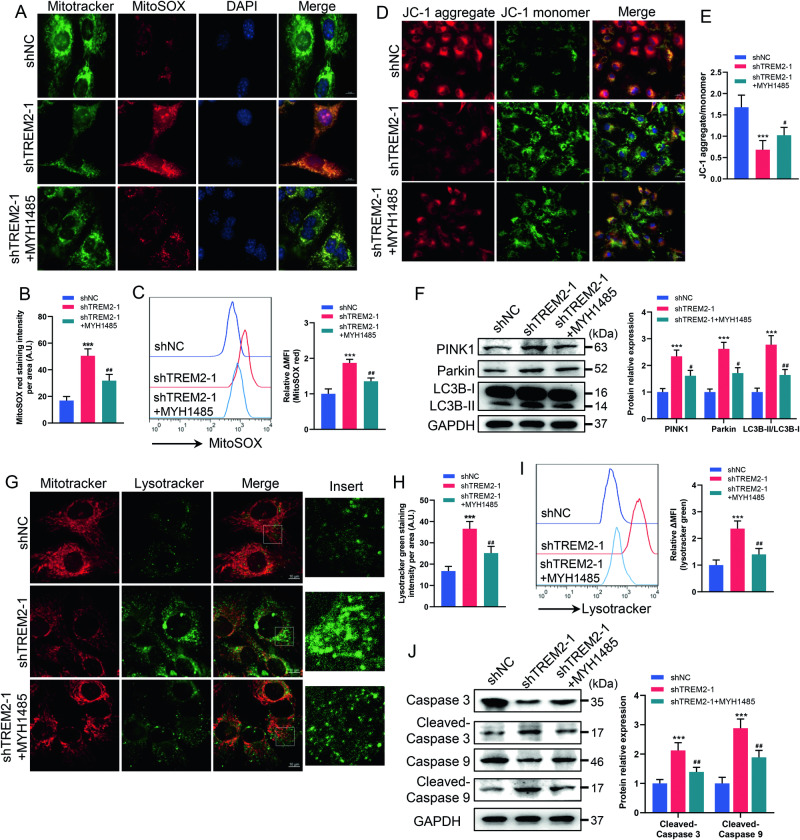
mTOR activator rescued mitochondrial damage and autophagy caused by TREM2 deficiency. TREM2-deficient SCs (shTREM2-1) were treated with the mTOR activator MHY1485 (5 μM). **A**, **B** Mitochondria were stained with MitoSOX Red and measured by immunofluorescence. Scale bars, 10 μm. *N* = 4, *n* ≥ 10 fields/group. **C** Mitochondria were stained with MitoSOX Red and measured by flow cytometry. *n* = 3. **D** The mitochondrial membrane potential (MMP) was measured by a JC-1 probe. Scale bars, 20 μm. **E** Quantification of the ratio of JC-1 aggregates/monomers. *N* = 4, *n* ≥ 10 fields/group. **F** The levels of PTEN-induced putative kinase 1 (PINK1) and parkin and the LC3BII/LC3BI ratio in SCs were determined by western blot. *n* = 3.**G**, **H** Lysosomes were stained with LysoTracker Green and measured by immunofluorescence. Scale bars, 10 μm. *N* = 4, *n* ≥ 10 fields/group. **I** Lysosomes were stained with LysoTracker Green and measured by flow cytometry. *n* = 3. **J** Levels of cleaved caspase 3, total caspase 3, cleaved caspase 9 and total caspase 9 were determined by western blot. *n* = 3. ***p < 0.001, compared with shNC; ^#^*p* < 0.05, ^##^*p* < 0.01, compared with shTREM2-1.

We conducted immunofluorescence staining and flow cytometry and found that treatment with MYH1485 reduced mitochondrial ROS production and partially reversed the impairment of the MMP in the TREM2 knockdown group (Fig. 7A–E). Western blot analysis showed that MYH1485 treatment decreased the expression levels of PINK1 and parkin and the LC3BII/LC3BI ratio in the TREM2 knockdown group, indicating that TREM2 deficiency reversed the activation of autophagy (Fig. 7F). These results were further confirmed by the reduction in lysosomes caused by MYH1485 treatment in the TREM2 knockdown group (Fig. 7G–I). Additionally, MYH1485 treatment partially reversed the mitochondria-dependent apoptosis activation caused by TREM2 deficiency, as evidenced by decreased levels of cleaved caspase-3 and caspase-9 (Fig. 7J). Taken together, these results demonstrate that TREM2 deficiency impairs energy metabolism and induces mitochondrial damage and autophagy by regulating PI3K-AKT-mTOR signaling in SCs.

### TREM2 deficiency exacerbates PNS damage in a mouse model of AMAN

**Fig. 8:**
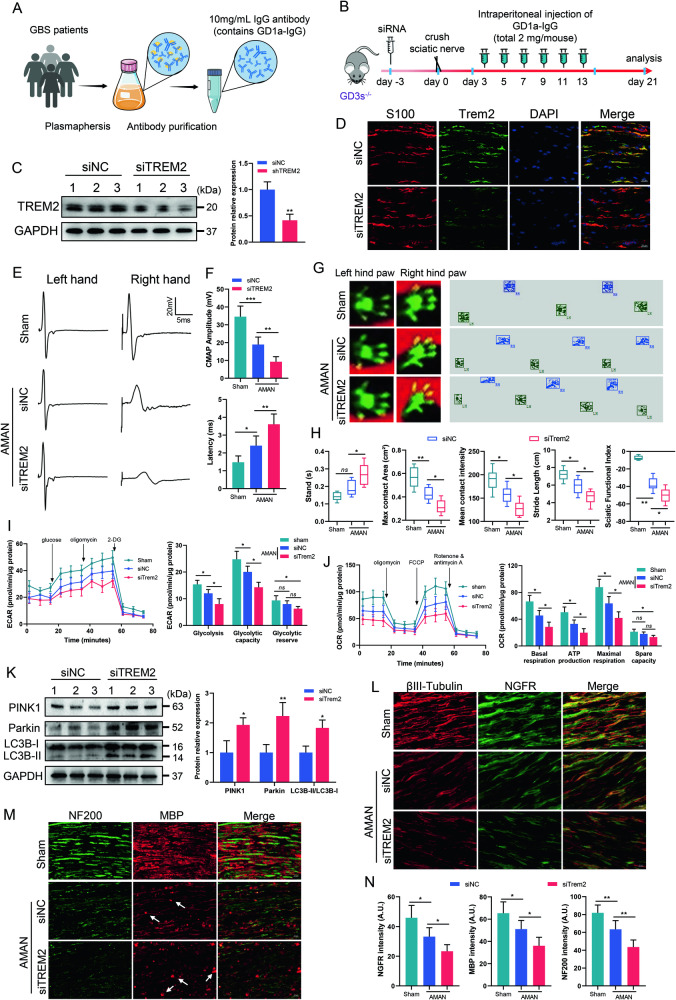
TREM2 deficiency exacerbates PNS damage in a mouse model of AMAN. **A** Schematic diagram of GD1a-IgG purification. **B** Schematic of the experimental process in the mouse model of AMAN. Negative control siRNA (siNC) or TREM2 siRNA (siTREM2) was injected in situ into the right sciatic nerve 3 days before surgery. Downregulated TREM2 expression was identified by western blot (**C**) and immunofluorescence staining (**D**). Scale bars, 20 μm. *n* = 3. **E**, **F** Electrophysiological tests were applied to evaluate nerve conduction in the sciatic nerve, and the complex muscle action potential (CMAP) amplitude and latency were quantified. *n* = 6. **G**, **H** Gait analysis was applied to evaluate motor function, and gait parameters and sciatic functional index were quantified. *n* = 6. **I**, **J** The right sciatic nerves were isolated and dissociated into a cell suspension. Extracellular acidification rate (ECAR) (**I**) and oxygen consumption rate (OCR) (**J**) were determined by Seahorse experiments. Quantitative analysis of glycolysis, glycolytic capacity and glycolytic reserve in the ECAR and basal respiration, ATP production, maximal respiration, and spare respiratory capacity in the OCR. *n* = 4. **K** The levels of PTEN-induced putative kinase 1 (PINK1) and parkin and the LC3BII/LC3BI ratio in sciatic nerves were determined by western blot. n = 3. **L**–**N** Representative immunofluorescence and quantification of nerve growth factor receptor (NGFR), Neurofilament (NF) 200 and myelin basic protein (MBP) in sciatic nerves. Arrows indicate myelin debris. As indicated, TREM2 deficiency significantly impaired the clearance of myelin debris (arrows) and inhibited nerve regeneration. Scale bar, 20 μm. *N* = 6, *n* ≥ 6 fields/group. *ns*, not significant, *p < 0.05, **p < 0.01.

To elucidate the supporting role of TREM2-regulated SCs energy metabolism in PNS, an axonal model of AMAN was established by intraperitoneal injection of GD1a-IgG and sciatic nerve crush in GD3s^−/−^ mice. SiRNA was injected locally 3 days prior to sciatic nerve crush (Fig. 8A, B). TREM2 expression was determined by western blot and immunofluorescence staining. The results showed a significant decrease in TREM2 expression in the siTREM2 group compared to that in the control group (Fig. 8C, D). Nerve conduction was evaluated using electrophysiological tests, while motor function was assessed using gait analysis. The results showed that TREM2 deficiency exacerbated the impairment in nerve conduction in AMAN mice, as evidenced by the reduced amplitude and increased latency of CMAP (Fig. 8E, F). Gait analysis demonstrated that TREM2 deficiency further worsened the compromised motor function in AMAN mice, reflected in increased stand time and reduced max contact area, mean contact intensity, stride length and sciatic functional index (Fig. 8G, H). To further examine the effect of TREM2 deficiency on the energy metabolism of the sciatic nerve in AMAN mice, the ECAR and OCR were measured to evaluate the glycolytic stress and mitochondrial respiratory capacity, respectively. The results showed that TREM2 deficiency exacerbated the impaired ECAR in AMAN mice, indicated by diminished glycolysis, glycolytic capacity and glycolytic reserve (Fig. 8I). OCR analysis demonstrated that TREM2 deficiency further impaired the mitochondrial respiratory capacity in AMAN mice, evident in the diminished basal respiration, ATP production, maximal respiration and spare capacity (Fig. 8J). The impairment of energy metabolism in sciatic nerves prompted us to explore its influence on mitochondrial autophagy. We found that TREM2 deficiency increased the levels of PINK1 and parkin and the LC3BII/LC3BI ratio, indicating accelerated autophagy (Fig. 8K). Moreover, immunofluorescence staining demonstrated that TREM2 deficiency further diminished the expression of NGFR, a marker for SCs with proposed regenerative potential, in the injured sciatic nerves of AMAN mice (Fig. 8L, N). Ultimately, TREM2 deficiency hindered the clearance of myelin debris, exacerbated myelin damage and further impaired support for axonal growth, resulting in decreased expression levels of neurofilament (NF) 200 and myelin basic protein (MBP) in the injured sciatic nerves of AMAN mice (Fig. 8M, N). In summary, TREM2 deficiency impaired the energy metabolism of the sciatic nerve and exacerbated neurological deficits, impairing nerve regeneration and functional recovery.

## Discussion

SCs serve as primary glial cells in the PNS, providing substantial material and energy support for neuronal growth [45]. Understanding the regulatory mechanisms that govern the energy metabolism of SCs is crucial for facilitating neuronal energy supply via glia-to-axon coupling during nerve repair following injury. In this study, we demonstrated that TREM2 deficiency impairs cell proliferation, glycolytic flux and oxidative metabolism in SCs. Importantly, the energy crisis caused by TREM2 deficiency activated AMPK and impaired PI3K-AKT-mTOR signaling and further triggered mitochondrial damage and autophagy. Furthermore, TREM2 deficiency impaired energy metabolism and axonal growth in the sciatic nerve, resulting in exacerbated neurological deficits and suppressed nerve regeneration in a mouse model of AMAN.

Previous research has predominantly focused on the expression and function of TREM2 in microglia, highlighting its crucial role in phagocytosis, inflammation, and the immune response in the CNS [1]. TREM2 deficiency has been implicated in various neurodegenerative diseases, particularly Alzheimer’s disease [2, 3]. Further investigations revealed that TREM2 deficiency in microglia disrupts lipid metabolism and impairs glucose uptake, suggesting its involvement in maintaining microglial homeostasis [42, 46]. Although several studies have hinted at a potential role for TREM2 in modulating the immune response in peripheral monocytes and macrophages [47, 48], its expression and function in the PNS, specifically in SCs, remain unclear. Our study provides evidence that TREM2 is expressed in SCs but not in neurons in the PNS and that its deficiency impairs SC proliferation by impairing SYK and downstream signaling.

In the process of nerve regeneration, SCs clear nerve fiber debris and undergo reprogramming and myelination to provide a favorable environment for nerve regeneration [49]. Similar to glia in the CNS, SCs provide pyruvate and lactate to neurons to counteract the energy depletion of injured axons by metabolic coupling between glia and axons [50]. These processes require considerable energy, motivating us to investigate the regulatory mechanisms of SCs metabolism. The energy metabolomics analysis demonstrated a remarkable decrease in the levels of glycolytic products, nucleotide and amino acid products in TREM2-deficient SCs, indicating impairment of glycolysis, OXPHOS, nucleotide metabolism, amino acid metabolism and lipid metabolism. Our experiments support these findings, revealing that TREM2 deficiency impairs cell proliferation, glycolytic flux and oxidative metabolism in SCs, indicating decreased energy metabolism. The decrease in the ECAR and OCR also confirmed the simultaneous impairment of glycolysis and OXPHOS.

The downregulation of GLUT1 and GLUT4 expression contributes to decreased glucose intake and impaired initiation of glycolysis. The decreased expression of enzymes involved in glycolysis and the downregulation of PFKFB3 indicate impaired glycolysis in TREM2-deficient SCs. Consequently, the production of glycolytic products such as pyruvate and lactate is decreased in TREM2-deficient SCs. Insufficient acetyl-CoA is metabolized into the TCA cycle in mitochondria, resulting in decreased levels of amino acid metabolism, lipid synthesis and OXPHOS. The downregulation of mitochondrial ETC complex expression may underlie the impairment of OXPHOS, as evidenced by decreased levels of NAD^+^ and impaired MMP. TFAM is essential for modulating the mtDNA copy number and mitochondrial encoded components of respiratory complexes [51]. Impaired mitochondrial activity is the inevitable result of decreased mtDNA copy number and mitochondrial number, length and area, which are regulated by the downregulation of TFAM.

The energy crisis resulting from TREM2 deficiency not only impairs SC proliferation but also causes damage to mitochondria. Stress conditions, such as mitochondrial damage, can markedly increase the production of mitochondrial ROS, which in turn contributes to mitochondrial damage and triggers neuronal death signaling [35, 52, 53]. Consistent with these findings, our research showed a decrease in the MMP along with elevated ROS levels in TREM2-deficient SCs. In cases where insults persist, damaged mitochondria are eliminated by mitophagy, a process that targets mitochondria for lysosomal degradation [54]. Recent evidence suggests that excessive ROS production induces autophagy [36]. STAT3 plays a critical role in mitochondrial respiration, ROS production and autophagy [36, 37]. Our experiments strongly supported the notion that TREM2 deficiency induces PINK1-parkin-mediated mitochondrial autophagy by activating STAT3, which was further confirmed by increased mumbers of lysosomes. Autophagy is considered a double-edged sword. Mitophagy has been shown to significantly reduce Aβ deposition and prevent cognitive impairment in an AD mouse model [55, 56]. However, Ulland et al. reported that increased autophagy in TREM2-deficient microglia impairs energetic and biosynthetic metabolism in an AD mouse model [42]. In our study, the impairment of energy metabolism caused by TREM2 deficiency led to persistent mitochondrial damage, thereby accelerating mitochondrial autophagy. Additionally, mitochondrial lesions cause a decrease in the MMP and further activate mitochondria-dependent apoptosis.

TREM2 emerges as an innate immune receptor that affects the metabolism of SCs through a unique mechanism involving the activation of the PI3K-AKT-mTOR signaling pathway, which supports long-term cell nutrition, survival and proliferation [4, 42]. The pathway and its downstream targets HIF1-α and c-MYC are intimately involved in the regulation of cellular energetic and biosynthetic metabolism [43, 50, 57]. Energy metabolomics analysis revealed that several altered metabolites and KEGG regulatory pathways were associated with PI3K-AKT-mTOR signaling. Among these pathways, the AMPK signaling pathway, PI3K-AKT signaling pathway, mTOR signaling pathway and HIF-1 signaling pathway were identified to be enriched. Our findings indicated that the activation of AMPK and defective PI3K-AKT-mTOR signaling in TREM2-deficient SCs were associated with impaired energy metabolism and mitochondrial autophagy. Defective mTOR signaling significantly impairs the activation of p70S6K, leading to the dysregulation of HIF-1α/c-MYC transcription and decreasing the expression of glycolytic enzymes, regulators of nucleotide and lipid synthesis. This inhibition further discourages protein synthesis, cell growth, and proliferation [50, 57, 58]. PI3K-AKT-mTOR signaling is recognized as a key upstream inhibitor of mitochondrial autophagy [59]. Our results indicated that impaired PI3K-AKT-mTOR signaling caused by TREM2 deficiency activates mitochondrial autophagy. Additionally, MYH1485 is an effective activator of mTOR signaling [60]. Our results demonstrated that MYH1485 treatment partially reversed the impaired glycolytic flux and oxidative metabolism while suppressing autophagy in TREM2-deficient SCs. Notably, the outcomes caused by TREM2 deficiency were not fully reversed by MYH1485, suggesting that TREM2 deficiency might induce alterations in other molecular signaling pathways in SCs. Several studies have reported that TREM2/DAP12 maintains metabolic homeostasis and regulates cell proliferation and survival by phosphorylating SYK and mediating downstream proline-rich tyrosine kinase 2 /β-catenin signaling and AMPK/UNC-51-like autophagy activated kinase 1 signaling in macrophages and microglia [42, 61]. However, further studies are warranted to elucidate the intricate molecular mechanisms involved. Altogether, these findings indicated that mTOR activation could rescue TREM2 deficiency by restoring energy metabolism and inhibiting autophagy activation.

Furthermore, we utilized intraperitoneal injection of GD1a-IgG and sciatic nerve crush injury to simulate the suppression of axonal regeneration by anti-ganglioside antibodies, allowing us to investigate the role of TREM2 in a mouse model of AMAN. Our findings showed that TREM2 deficiency significantly disrupts glycolytic flux and oxidative metabolism in the injured sciatic nerve. We observed PINK1-parkin-mediated autophagy activation in TREM2-deficient injured sciatic nerves, indicating severe mitochondrial damage. The energy crisis caused by TREM2 deficiency markedly exacerbates myelin damage and further impairs support for axonal growth, exacerbating PNS damage and impairing regeneration and functional recovery. This was evidenced by the low expression levels of NGFR, NF200 and MBP. Taken together, the results of our study highlight the critical role of TREM2 in maintaining the energy metabolism of SCs, suggesting that strategies aimed at sustaining SC metabolism could be promising for therapeutic intervention in peripheral nerve injury.

### Supplementary information

Supplementary materials

Supplement figure 1

Supplement figure 2

Supplement figure 3

Supplement figure 4

Supplement figure 5

Supplement figure 6

aj-checklist

Original Data File

## Data Availability

The data that support the findings of this study are available from the corresponding author upon reasonable request.
