# Supplementary material for: TREM2 deficiency impairs the energy metabolism of Schwann cells and exacerbates peripheral neurological deficits
Source: Cell Death Dis. 2024 Mar 7;15(3):193. doi: 10.1038/s41419-024-06579-9 (PMC10920707; doi:10.1038/s41419-024-06579-9)
Supplement: Supplementary file 1 — Supplementary materials [file 41419_2024_6579_MOESM1_ESM.docx]

**Supplemental materials**

**Supplemental table 1. Primer sequences for qRT‒qPCR.**

| Gene |  | Primer sequence (5’-3’) |
| --- | --- | --- |
| TREM2 | Forward | CTGGAACCGTCACCATCACTC |
|  | Reverse | CGAAACTCGATGACTCCTCGG |
| GLUT1 | Forward | CAGTTCGGCTATAACACTGGTG |
|  | Reverse | GCCCCCGACAGAGAAGATG |
| GLUT4 | Forward | GGACCGGATTCCATCCCAC |
|  | Reverse | TCCCAACCATTGAGAAATGATGC |
| HK2 | Forward | ATGATCGCCTGCTTATTCACG |
|  | Reverse | CGCCTAGAAATCTCCAGAAGGG |
| PFKFB3 | Forward | CAACTCCCCAACCGTGATTGT |
|  | Reverse | GAGGTAGCGAGTCAGCTTCTT |
| PKM2 | Forward | GTGCCGCCTGGACATTGACTC |
|  | Reverse | ATTCAGCCGAGCCACATTCATCC |
| LDHA | Forward | TCGCACCTTGTAGCCGTTATTGG |
|  | Reverse | ACTGCCCTCCCGCTCTTCTC |
| COX2 | Forward | ATAACCGAGTCGTTCTGCCAAT |
|  | Reverse | TTTCAGAGCATTGGCCATAGAA |
| Rsp18 | Forward | GTGTTAGGGGACTGGTGGACA |
|  | Reverse | CATCACCCACTTACCCCCAAA |
| mt-ND2 | Forward | ACCAAATCTCTCCCTCACTAAACG |
|  | Reverse | CCACCTCAACTGCCTGCTATG |
| SDHA | Forward | TTACAAAGTGCGGGTCGATGA |
|  | Reverse | TGTTCCCCAAACGGCTTCTT |
| mt-CYTB | Forward | CCCACCCCATATTAAACCCG |
|  | Reverse | GAGGTATGAAGGAAAGGTATAAGGG |
| mt-CO1 | Forward | TCCCAGATATAGCATTCCCACG |
|  | Reverse | ACTGTTCATCCTGTTCCTGC |
| mt-ATP5a1 | Forward | TCTCCATGCCTCTAACACTCG |
|  | Reverse | CCAGGTCAACAGACGTGTCAG |
| TFAM | Forward | ATTCCGAAGTGTTTTTCCAGCA |
|  | Reverse | TCTGAAAGTTTTGCATCTGGGT |
| GAPDH | Forward | AACGACCCCTTCATTGAC |
|  | Reverse | TCCACGACATACTCAGCAC |

**Supplemental table 2. Details of primary and secondary antibodies**

| Antibodies | Cat. number | Applications | Source |
| --- | --- | --- | --- |
| Rabbit anti-TREM2 | 27599-1-AP | WB: 1:1000  IF: 1:200 | Proteintech |
| Mouse anti-S100β | MA5-12969 | IF: 1:200 | Invitrogen |
| Mouse anti-TuJ1 | 66375-1-Ig | IF: 1:200 | Proteintech |
| Rabbit anti-SYK | 13198 | WB: 1:1000 | Cell Signal Technology |
| Rabbit anti-p-SYK (Tyr525/526) | 2710 | WB: 1:1000 | Cell Signal Technology |
| Rabbit anti-GLUT1 | ab115730 | WB: 1:500 IF: 1:200 | Abcam |
| Mouse anti-GLUT4 | BF1001 | WB: 1:500 IF: 1:200 | Affinity |
| Rabbit anti-PFKFB3 | ab181861 | WB: 1:1000 IF: 1:200 | Abcam |
| Rabbit anti-LDHA | DF6280 | WB: 1:1000 IF: 1:200 | Affinity |
| Rabbit anti-HK2 | DF6176 | WB: 1:2000 IF: 1:400 | Affinity |
| Rabbit anti-PKM2 | AF5234 | WB: 1:1000 IF: 1:200 | Affinity |
| Rabbit anti-mt-ND2 | A17968 | WB: 1:500 IF: 1:100 | ABclonal |
| Rabbit anti-SDHA | 14865-1-AP | WB: 1:1000 IF: 1:200 | Proteintech |
| Rabbit anti-mt-CYTB | 55090-1-AP | WB: 1:1000 IF: 1:200 | Proteintech |
| Rabbit anti-mt-CO1 | A7341 | WB: 1:500 IF: 1:100 | ABclonal |
| Rabbit anti-mt-ATP5a1 | 14676-1-AP | WB: 1:2000 IF: 1:400 | Proteintech |
| Rabbit anti-TFAM | 22586-1-AP | WB: 1:5000 IF: 1:500 | Proteintech |
| Rabbit anti-STAT3 | 12640 | WB: 1:1000 | Cell Signal Technology |
| Rabbit anti-p-STAT3 (Tyr705) | 9145 | WB: 1:1000 | Cell Signal Technology |
| Rabbit anti-PINK1 | DF7742 | WB: 1:1000 IF: 1:200 | Affinity |
| Rabbit anti-Parkin | sc-32282 | WB: 1:200 | Santacruz |
| Rabbit anti-LC3B | AF4650 | WB: 1:1000 IF: 1:200 | Affinity |
| Rabbit anti-Caspase-3 | 9962 | WB: 1:1000 | Cell Signal Technology |
| Rabbit anti-Cleaved Caspase-3 | 9661 | WB: 1:1000 | Cell Signal Technology |
| Mouse anti-Caspase-9 | 9508 | WB: 1:1000 | Cell Signal Technology |
| Rabbit anti-Cleaved Caspase-9 | 9505 | WB: 1:1000 | Cell Signal Technology |
| Rabbit anti-AMPKα1+ AMPKα2 | ab207442 | WB: 1:1000 | Abcam |
| Rabbit anti-p- AMPKα1(Thr183) + AMPKα2(Thr172) | ab133448 | WB: 1:1000 | Abcam |
| Rabbit anti-PI3K p85α | AF6241 | WB: 1:1000 | Affinity |
| Rabbit anti-p-PI3K p85α (Tyr607) | AF3241 | WB: 1:500 | Affinity |
| Rabbit anti-AKT | AF6261 | WB: 1:1000 | Affinity |
| Rabbit anti-p-AKT (Ser473) | AF0016 | WB: 1:500 | Affinity |
| Rabbit anti-mTOR | AF6308 | WB: 1:1000 | Affinity |
| Rabbit anti-p-mTOR (Ser2448) | 5536 | WB: 1:1000 | Cell Signal Technology |
| Rabbit anti-p70S6K | AF6226 | WB: 1:1000 | Affinity |
| Rabbit anti-p-p70S6K (Thr389/Thr412) | AF3228 | WB: 1:500 | Affinity |
| Rabbit anti-HIF-1α | 20960-1-AP | WB: 1:2000 | Proteintech |
| Rabbit anti-c-MYC | 10828-1-AP | WB: 1:2000 | Proteintech |
| Mouse anti-β-Tubulin | MA1-118 | IF: 1:500 | Invitrogen |
| Rabbit anti-NGFR | PA5-27656 | IF: 1:200 | Invitrogen |
| Rabbit anti-NF200 | ab8135 | IF: 1:200 | Abcam |
| Rat anti-MBP | MAB386 | IF: 1:200 | Sigma |
| Mouse anti-GAPDH | AC033 | WB: 1:10000 | ABclonal |
| Goat anti-mouse IgG-HRP | AS064 | WB: 1:5000 | ABclonal |
| Goat anti-Rabbit IgG-HRP | AS063 | WB: 1:5000 | ABclonal |
| Goat anti-mouse IgG, Alexa Fluor™ 488 | A32723 | IF: 1:500 | Invitrogen |
| Donkey anti-Mouse IgG, Alexa Fluor™ 555 | A31570 | IF: 1:500 | Invitrogen |
| Goat anti-mouse IgG2a, Alexa Fluor™ 555 | A21137 | IF: 1:500 | Invitrogen |
| Goat anti-rabbit IgG, Alexa Fluor™ 488 | A-11034 | IF: 1:500 | Invitrogen |
| Donkey Anti-Rat IgG, Alexa Fluor™ 555 | ab150154 | IF: 1:500 | Abcam |

**Supplemental figure legends**

**Figure S1. Primary SCs were immunostainind for S100β (green) and NGFR (red)**

As shown, almost all primary SCs were positive for S100β and NGFR immunostaining. Scale bar: 50 μm.

**Figure S2. Targeted energy metabolomics analysis of energy-related metabolite changes in TREM2-deficient SCs.**

(A). Hierarchical clustering analysis of energy-related metabolites detected in SCs. Hierarchical clustering analysis was performed based on the normalized peak intensities of the intracellular metabolites. Rows represent individual metabolites. The columns represent individual replicates, with a total of 6 replicates per group. For each metabolite, the colors represent values of the relative metabolite abundance normalized in a range between -4 (blue = low abundance) and 4 (red = high abundance). (B). Principal component analysis (PCA) score plot. (C). Partial least squares-discriminant analysis (OPLS-DA) score plot. (D). Correlation analyses of the differentially abundant metabolites were performed to evaluate the metabolic proximities among significantly different metabolites. The line represents the correlation value of metabolites at the corresponding position.

**Figure S3. Regulatory interaction network analysis based on differentially abundant metabolites induced by TREM2 deficiency.**

The red dots represent a metabolic pathways, the yellow dots represent the regulatory enzymes related to a substance, the green dots represent the background substances of a metabolic pathway, the purple dots represent the molecular modules of a class of substances, the blue dots represent the chemical interaction reactions of a substance, and the green squares represent the differential substances obtained by this comparison.

**Figure S4. TREM2 deficiency promotes mitochondrial autophagy in SCs.**

(A). Mitochondria were stained with MitoSOX Red and measured by flow cytometry. n = 3. (B-C). Representative immunofluorescence images of PTEN-induced putative kinase 1 (PINK1) and LC3B in SCs. As indicated, PINK1^+^ and LC3B^+^ autophagosomes accumulated in TREM2-deficient SCs. (D). Lysosomes were stained with LysoTracker Green and measured by flow cytometry. n = 3. ****p* < 0.001.

**Figure S5. mTOR activator rescues impaired glycolytic flux and oxidative metabolism caused by TREM2 deficiency**

TREM2-deficient SCs (shTREM2-1) were treated with the mTOR activator MHY1485 (5 μM). (A). qRT‒PCR analysis of glycolysis-related mRNAs in SCs, including glucose transporter 1/4 (GLUT)1/4, hexokinase 2 (HK2), 6-phosphofructo-2-kinase/fructose-2,6-bisphosphatase 3 (PFKFB3), pyruvate kinase 2 (PKM2) and lactate dehydrogenase A (LDHA). n=3 per group. (B-E). Glucose uptake was measured by flow cytometry using 2-[N-(7-nitrobenz-2-oxa-1,3- diazol-4-yl)amino]-2-deoxy-D-glucose (2-NBDG) (B). Intracellular levels of glucose-6-phosphate (G6P) (C), pyruvate (D) and lactate (E) were measured by chemiluminescence. n = 4. (F). The mitochondrial DNA (mtDNA) copy number was measured by qRT‒PCR. (G-H). qRT‒PCR analysis of the mRNA expression of transcription factor A (TFAM) and mitochondrial electron transport chain (ETC) components, including mitochondrially encoded NADH dehydrogenase 2 (mt-ND2), succinate dehydrogenase complex flavoprotein subunit A (SDHA), mitochondrially encoded cytochrome b (mt-CYTB), mitochondrially encoded cytochrome C oxidase I (mt-CO1), and mitochondrially encoded ATP synthase alpha-subunit (mt-ATP5a1). n=3 per group. (I-K). Intracellular levels of ATP production (I), nicotinamide adenine dinucleotide (NAD^+^) (J) and the NAD^+^/NADH ratio (K) were measured by chemiluminescence. n = 4. ***p* < 0.01, ****p* < 0.001, compared with shNC; ^#^*p* < 0.05, ^##^*p* < 0.01, compared with shTREM2-1.

**Figure S6. mTOR activator rescues mitochondrial damage caused by TREM2 deficiency**

TREM2-deficient SCs (shTREM2-1) were treated with the mTOR activator MHY1485 (5 μM). (A-B). Representative fluorescence images and quantification of mitochondria in SCs stained with MitoTracker Green. Scale bars, 10 μm. N = 4, n ≥ 10 fields/group. (C-D). Representative electron microscopy images and quantification of mitochondrial length and area in SCs. Scale bars, 1 μm. N = 6, n ≥ 10 fields/group. ****p* < 0.001, compared with shNC; ^#^*p* < 0.05, ^##^*p* < 0.01, compared with shTREM2-1.
