# Supplementary figures and images for: TREM2 deficiency impairs the energy metabolism of Schwann cells and exacerbates peripheral neurological deficits
Source: Cell Death Dis. 2024 Mar 7;15(3):193. doi: 10.1038/s41419-024-06579-9 (PMC10920707; doi:10.1038/s41419-024-06579-9)

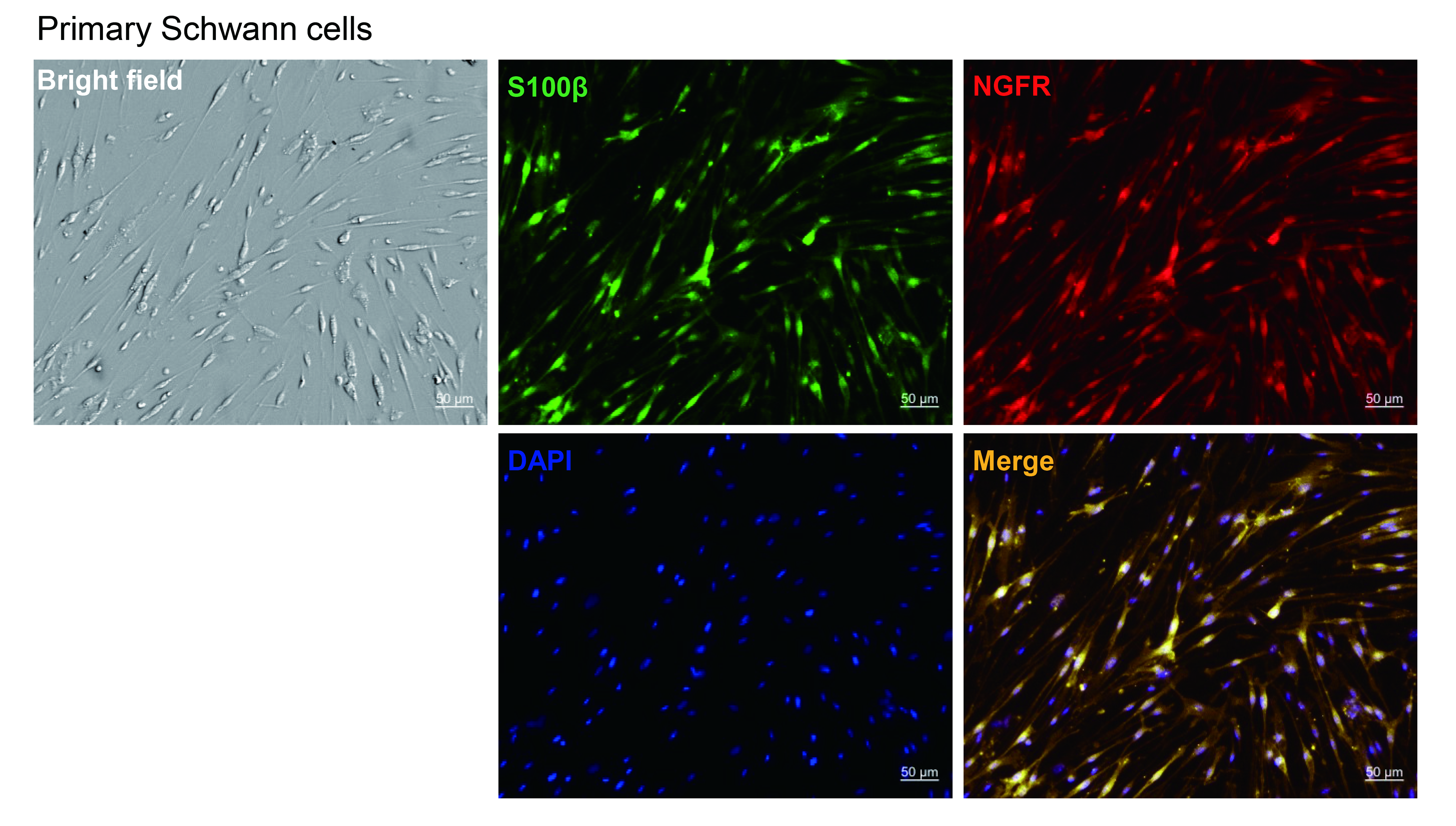

Supplement: Supplementary file 2 — Supplement figure 1 [file 41419_2024_6579_MOESM2_ESM.tif]

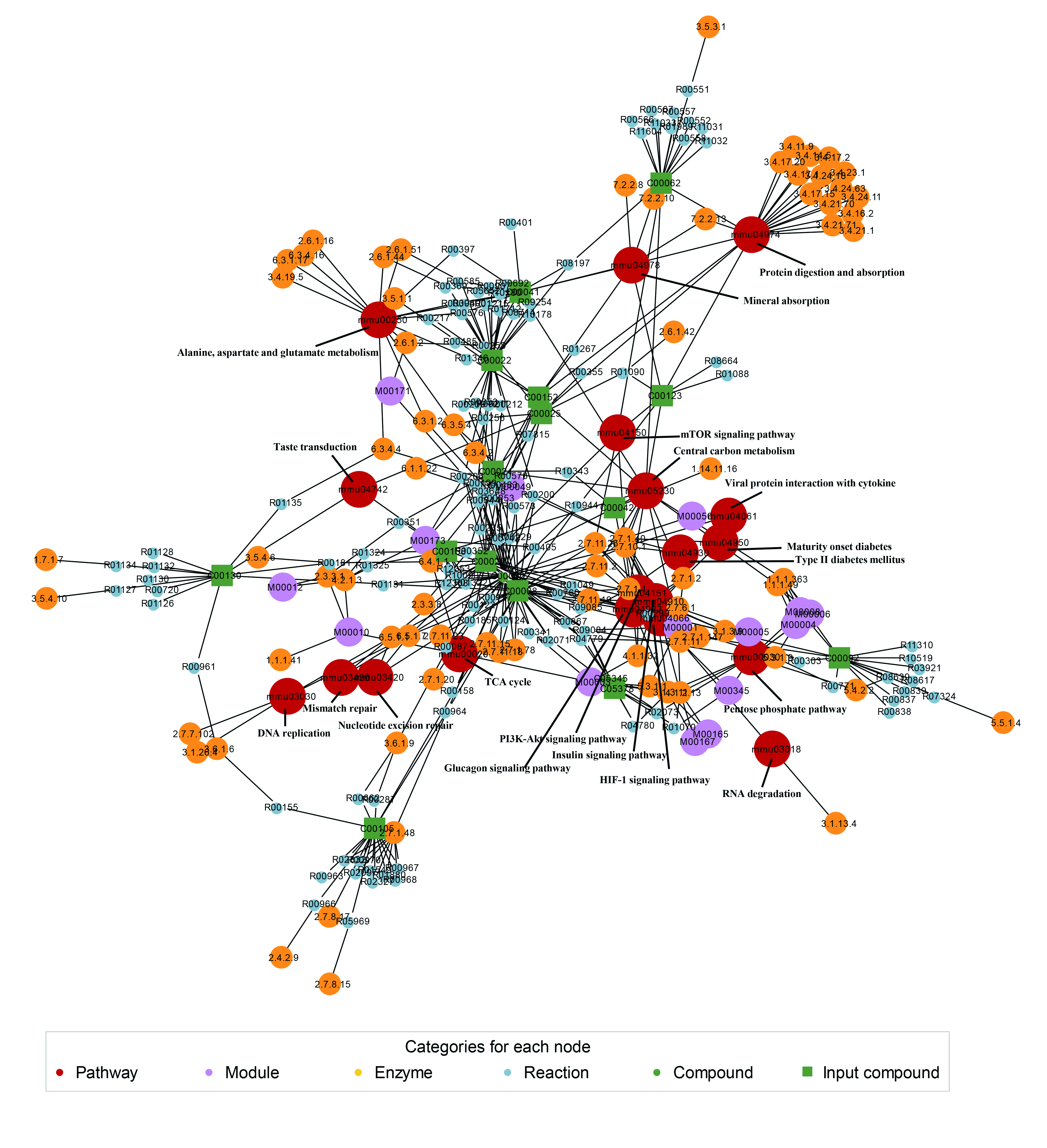

Supplement: Supplementary file 4 — Supplement figure 3 [file 41419_2024_6579_MOESM4_ESM.tif]

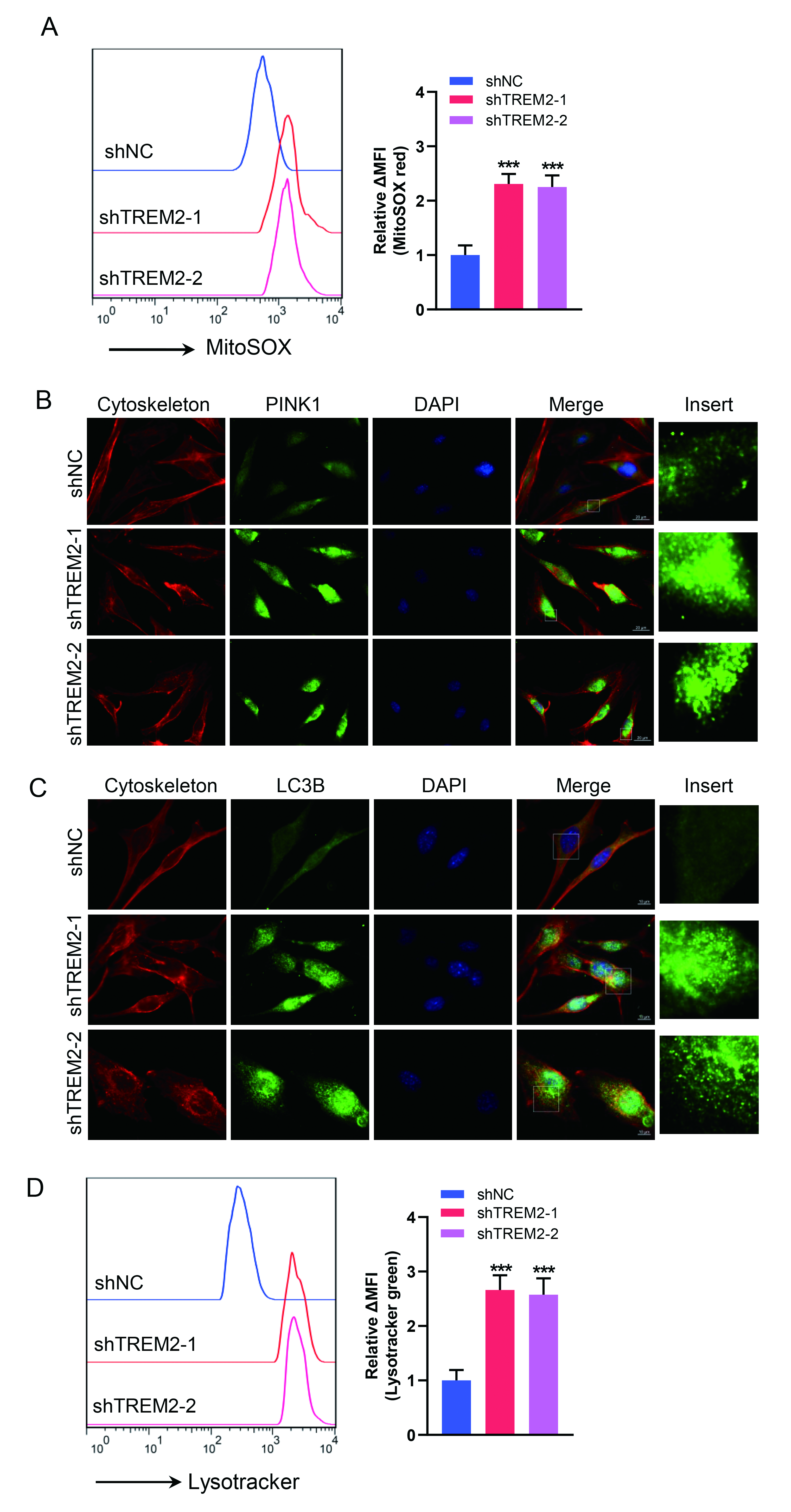

Supplement: Supplementary file 5 — Supplement figure 4 [file 41419_2024_6579_MOESM5_ESM.tif]

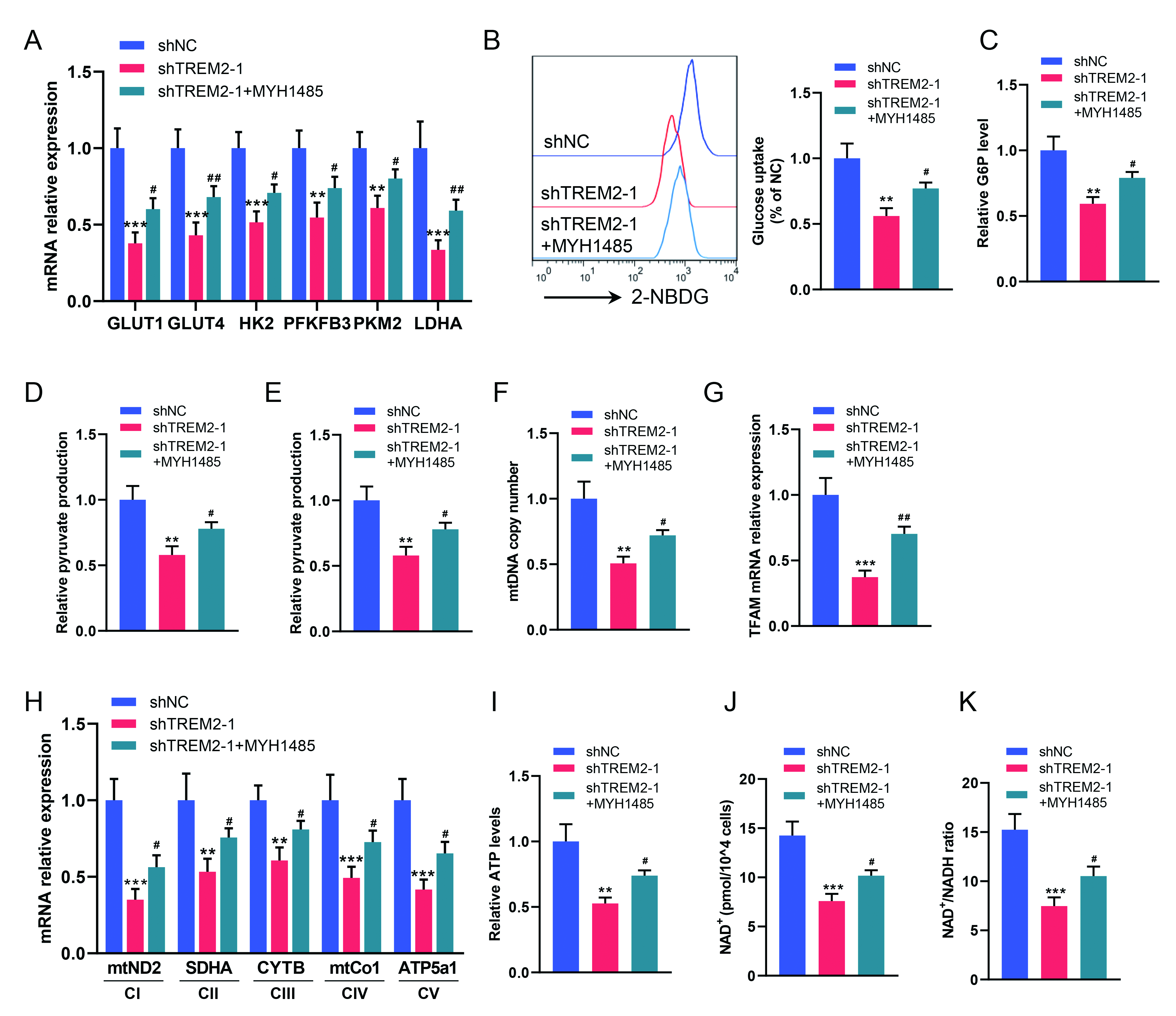

Supplement: Supplementary file 6 — Supplement figure 5 [file 41419_2024_6579_MOESM6_ESM.tif]

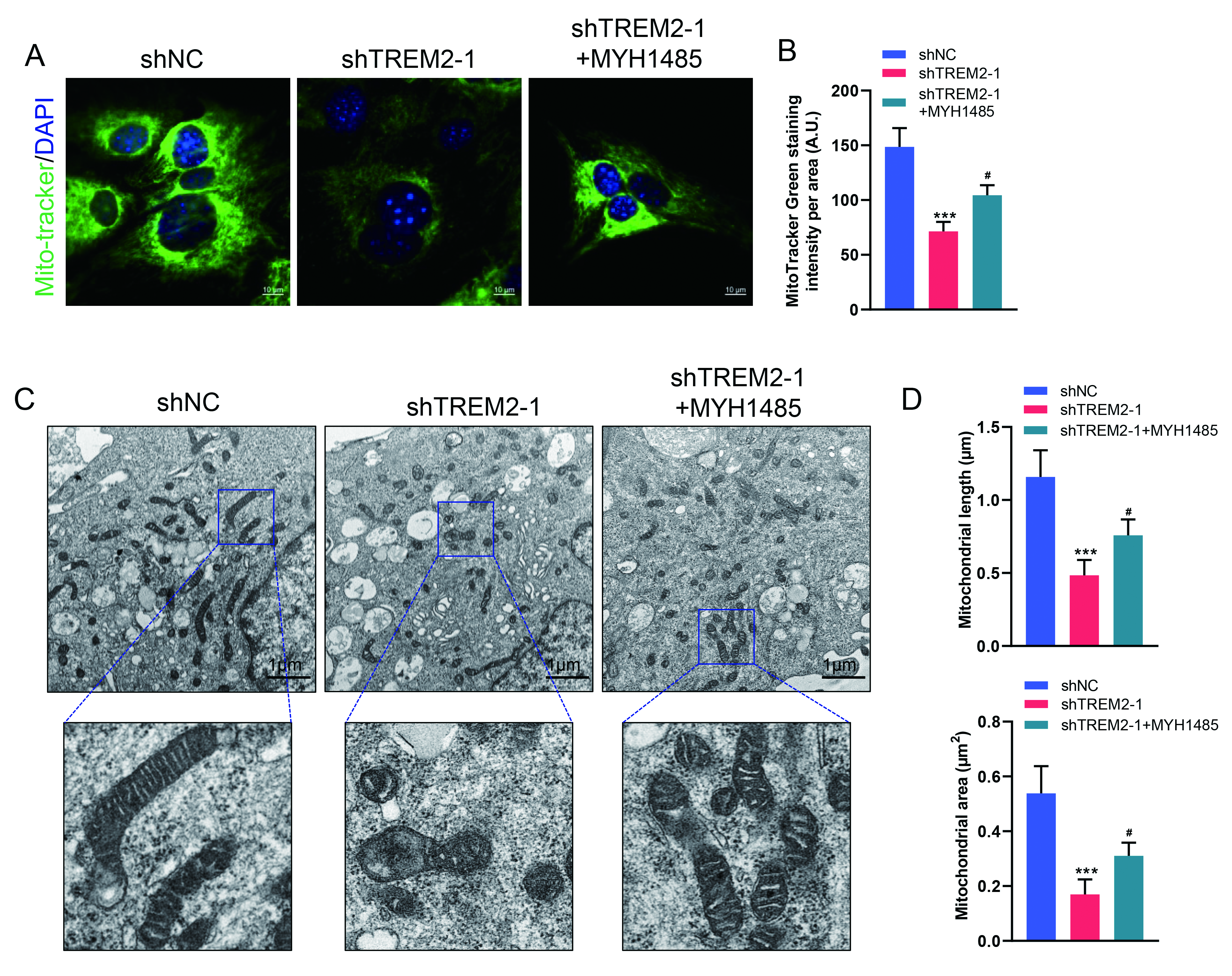

Supplement: Supplementary file 7 — Supplement figure 6 [file 41419_2024_6579_MOESM7_ESM.tif]
