# Supplementary material for: TREM2 deficiency impairs the energy metabolism of Schwann cells and exacerbates peripheral neurological deficits
Source: Cell Death Dis. 2024 Mar 7;15(3):193. doi: 10.1038/s41419-024-06579-9 (PMC10920707; doi:10.1038/s41419-024-06579-9)
Supplement: Supplementary file 9 — Original Data File [file 41419_2024_6579_MOESM9_ESM.pptx]

## Slide 1
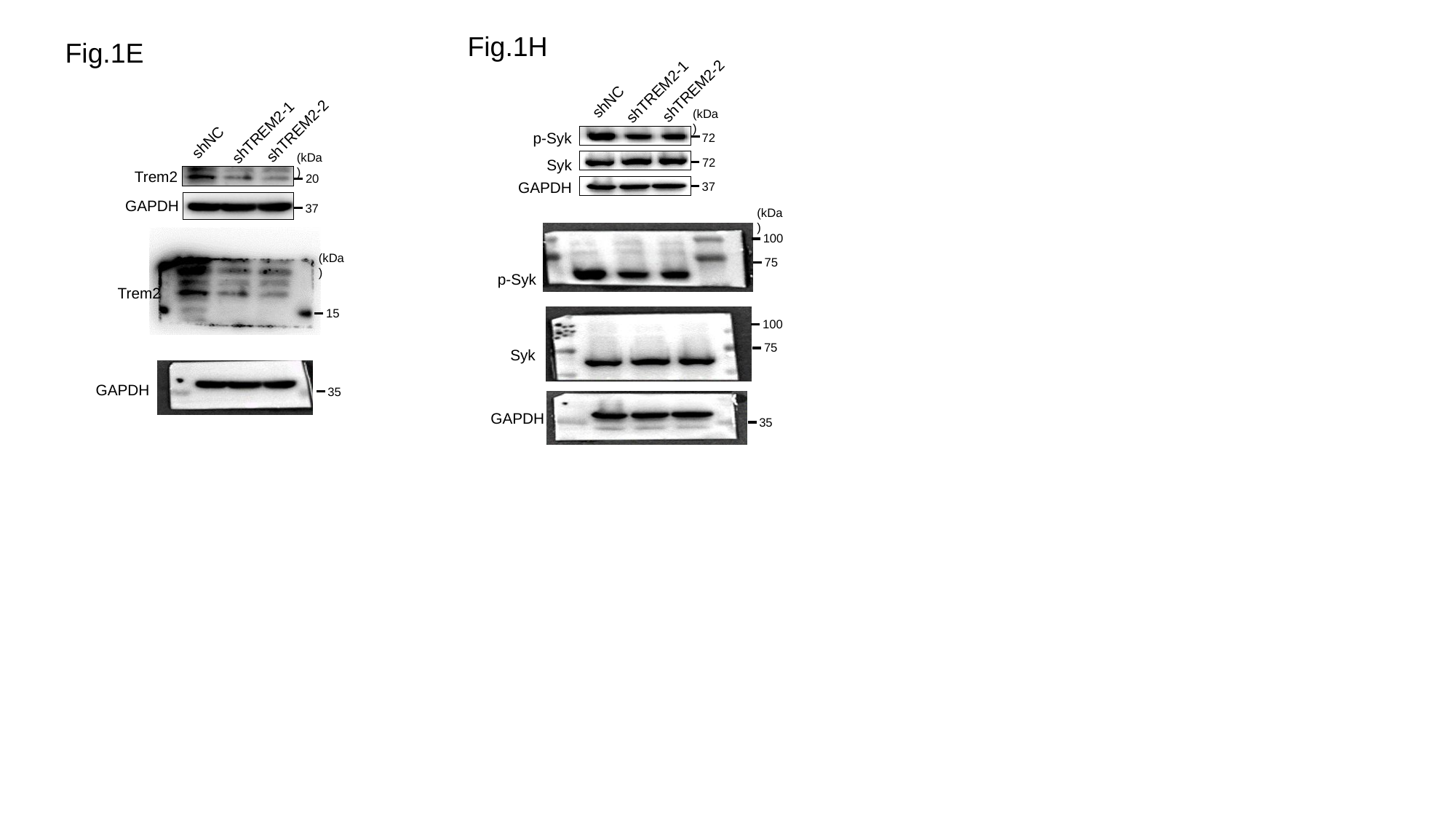

Fig.1H
Fig.1E
shTREM2-2
shTREM2-1
shNC
(kDa)
shTREM2-2
shTREM2-1
p-Syk
72
shNC
(kDa)
72
Syk
Trem2
20
GAPDH
37
GAPDH
37
(kDa)
100
(kDa)
75
p-Syk
Trem2
15
100
75
Syk
GAPDH
35
GAPDH
35

## Slide 2
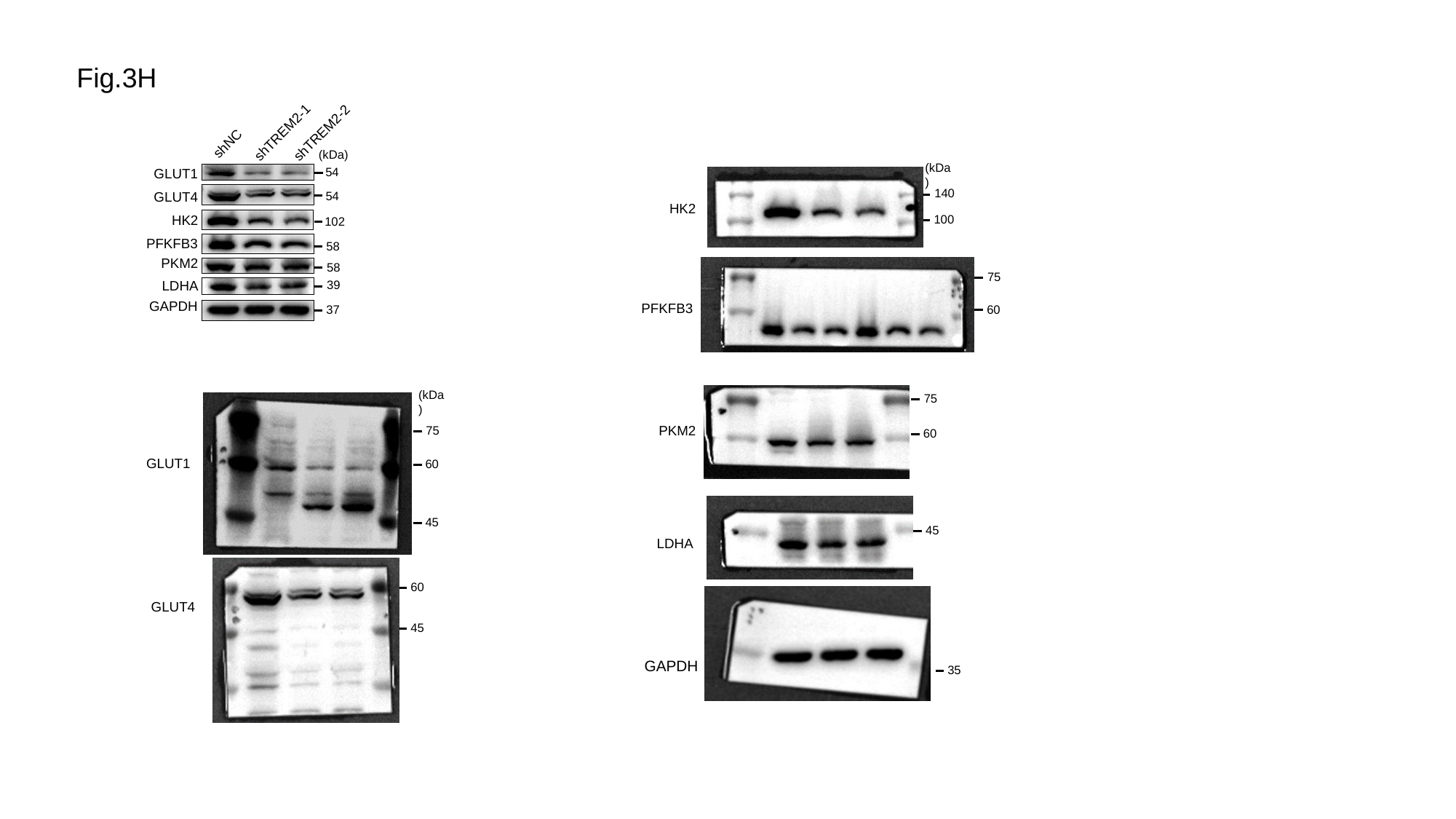

Fig.3H
shTREM2-1
shTREM2-2
shNC
(kDa)
54
GLUT1
54
GLUT4
HK2
102
PFKFB3
58
PKM2
58
LDHA
39
GAPDH
37
(kDa)
140
HK2
100
75
PFKFB3
60
(kDa)
75
PKM2
75
60
GLUT1
60
45
45
LDHA
60
GLUT4
45
GAPDH
35

## Slide 3
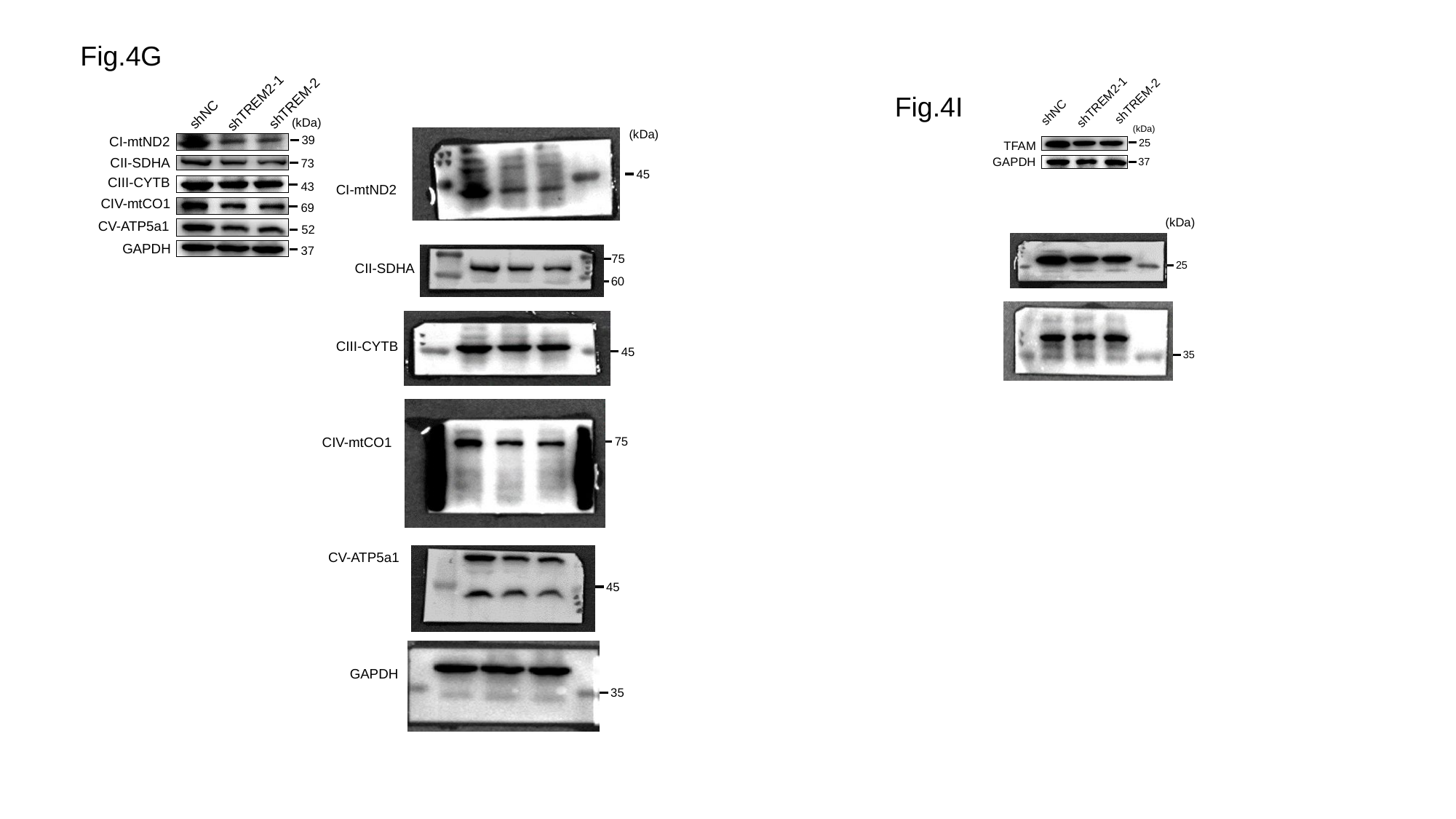

Fig.4G
Fig.4I
shTREM-2
shTREM2-1
shTREM2-1
shTREM-2
shNC
shNC
(kDa)
(kDa)
(kDa)
39
CI-mtND2
25
TFAM
CII-SDHA
GAPDH
37
73
45
CIII-CYTB
43
CI-mtND2
CIV-mtCO1
69
(kDa)
CV-ATP5a1
52
GAPDH
37
75
25
CII-SDHA
60
CIII-CYTB
45
35
CIV-mtCO1
75
CV-ATP5a1
45
GAPDH
35

## Slide 4
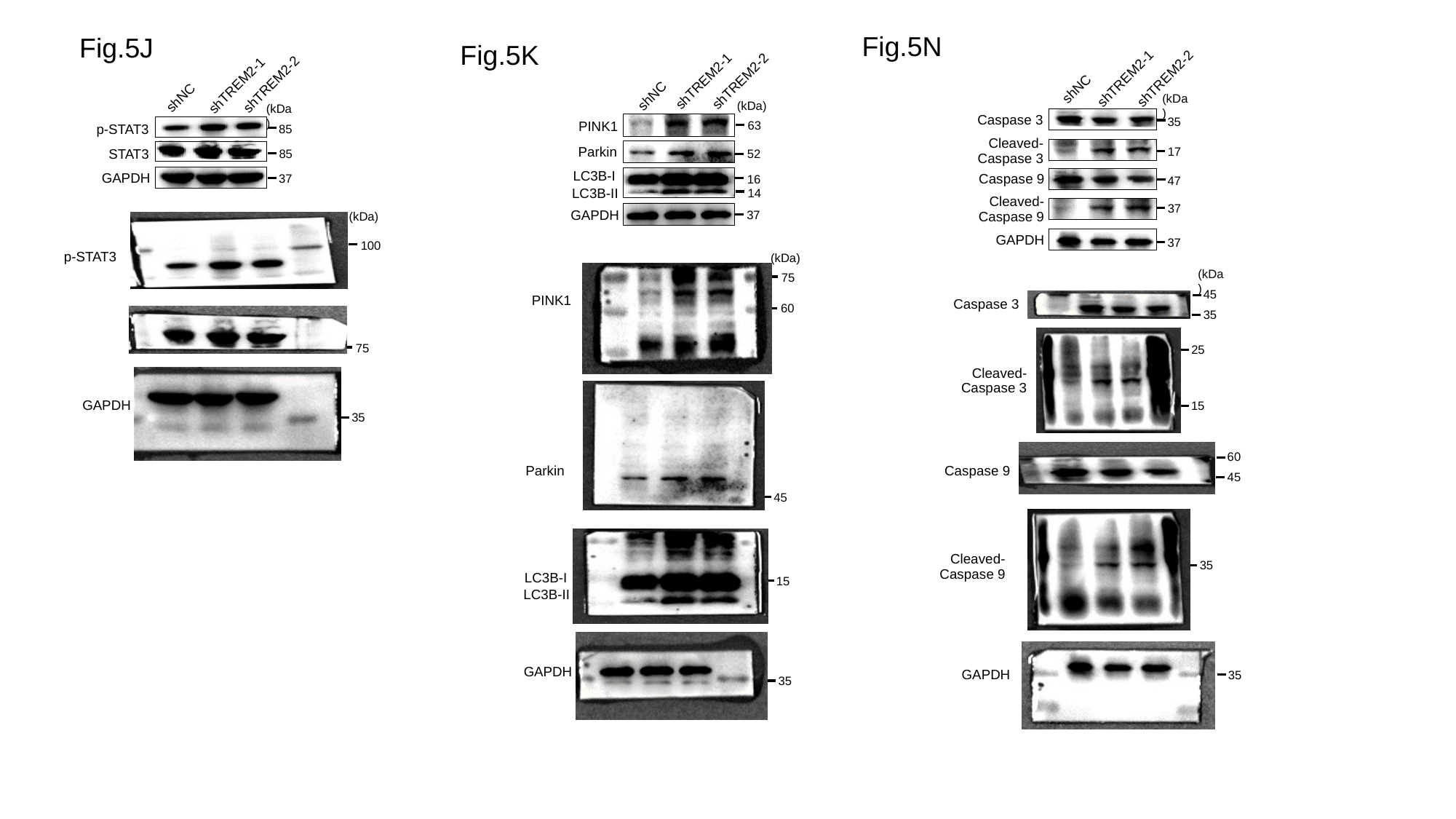

Fig.5N
Fig.5J
Fig.5K
shTREM2-2
shTREM2-1
shTREM2-1
shTREM2-2
shTREM2-2
shTREM2-1
shNC
shNC
shNC
(kDa)
(kDa)
(kDa)
Caspase 3
35
PINK1
63
p-STAT3
85
Cleaved-
Caspase 3
Parkin
17
STAT3
85
52
LC3B-I
GAPDH
Caspase 9
37
16
47
LC3B-II
14
Cleaved-
Caspase 9
37
GAPDH
37
(kDa)
GAPDH
37
100
p-STAT3
(kDa)
(kDa)
75
45
PINK1
Caspase 3
60
35
75
25
Cleaved-
Caspase 3
GAPDH
15
35
60
Parkin
Caspase 9
45
45
Cleaved-
Caspase 9
35
LC3B-I
15
LC3B-II
GAPDH
GAPDH
35
35

## Slide 5
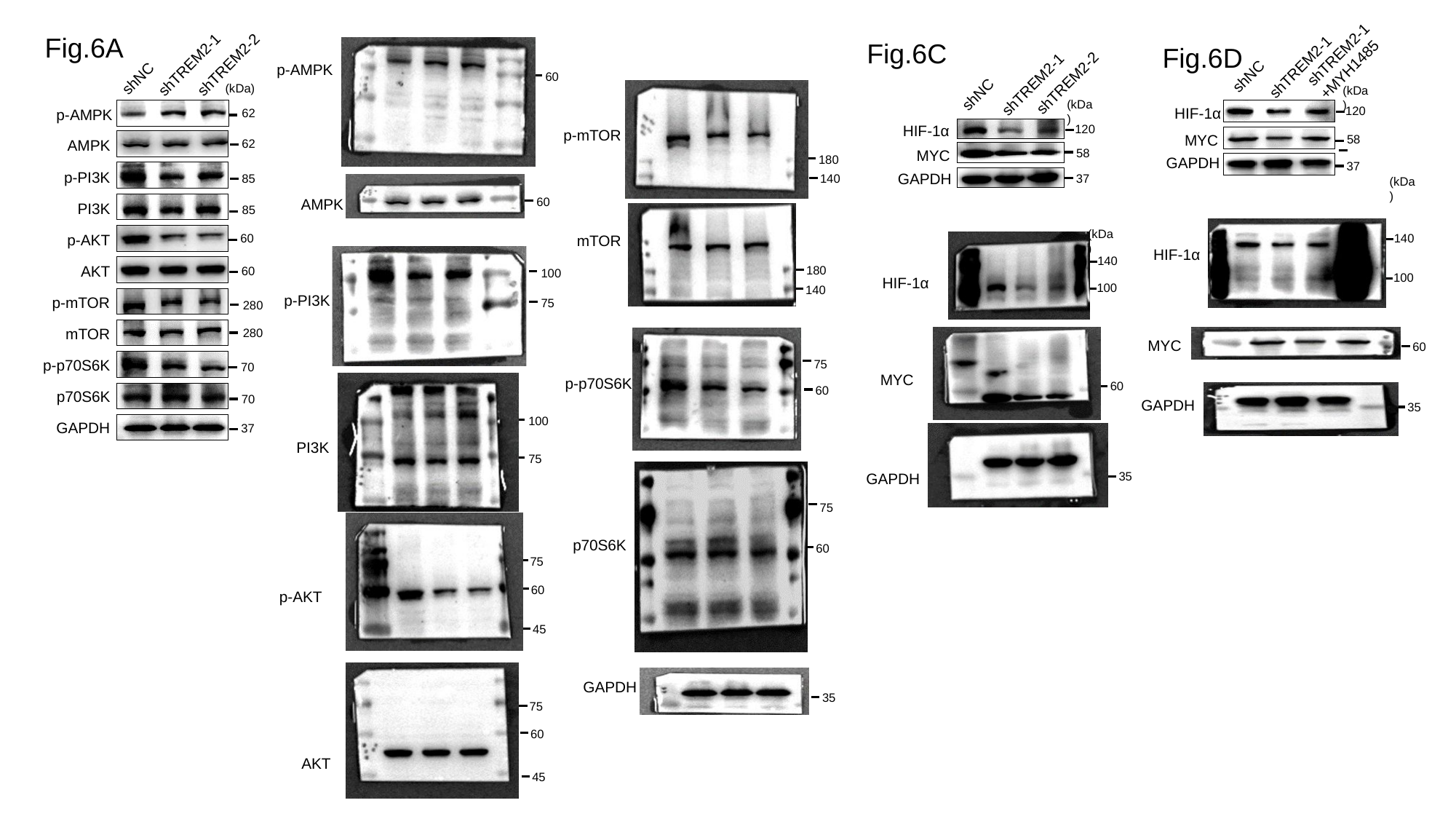

Fig.6A
Fig.6C
Fig.6D
shTREM2-1
+MYH1485
shTREM2-1
shTREM2-2
shTREM2-1
p-AMPK
shNC
shNC
60
shTREM2-2
shTREM2-1
(kDa)
(kDa)
shNC
(kDa)
120
HIF-1α
p-AMPK
62
HIF-1α
120
p-mTOR
MYC
58
62
AMPK
58
MYC
180
GAPDH
37
p-PI3K
GAPDH
37
85
140
(kDa)
60
AMPK
PI3K
85
(kDa)
p-AKT
60
140
mTOR
HIF-1α
140
AKT
180
60
100
100
HIF-1α
100
140
p-PI3K
p-mTOR
75
280
mTOR
280
MYC
60
p-p70S6K
75
70
MYC
p-p70S6K
60
60
p70S6K
70
GAPDH
35
100
GAPDH
37
PI3K
75
GAPDH
35
75
p70S6K
60
75
60
p-AKT
45
GAPDH
35
75
60
AKT
45

## Slide 6
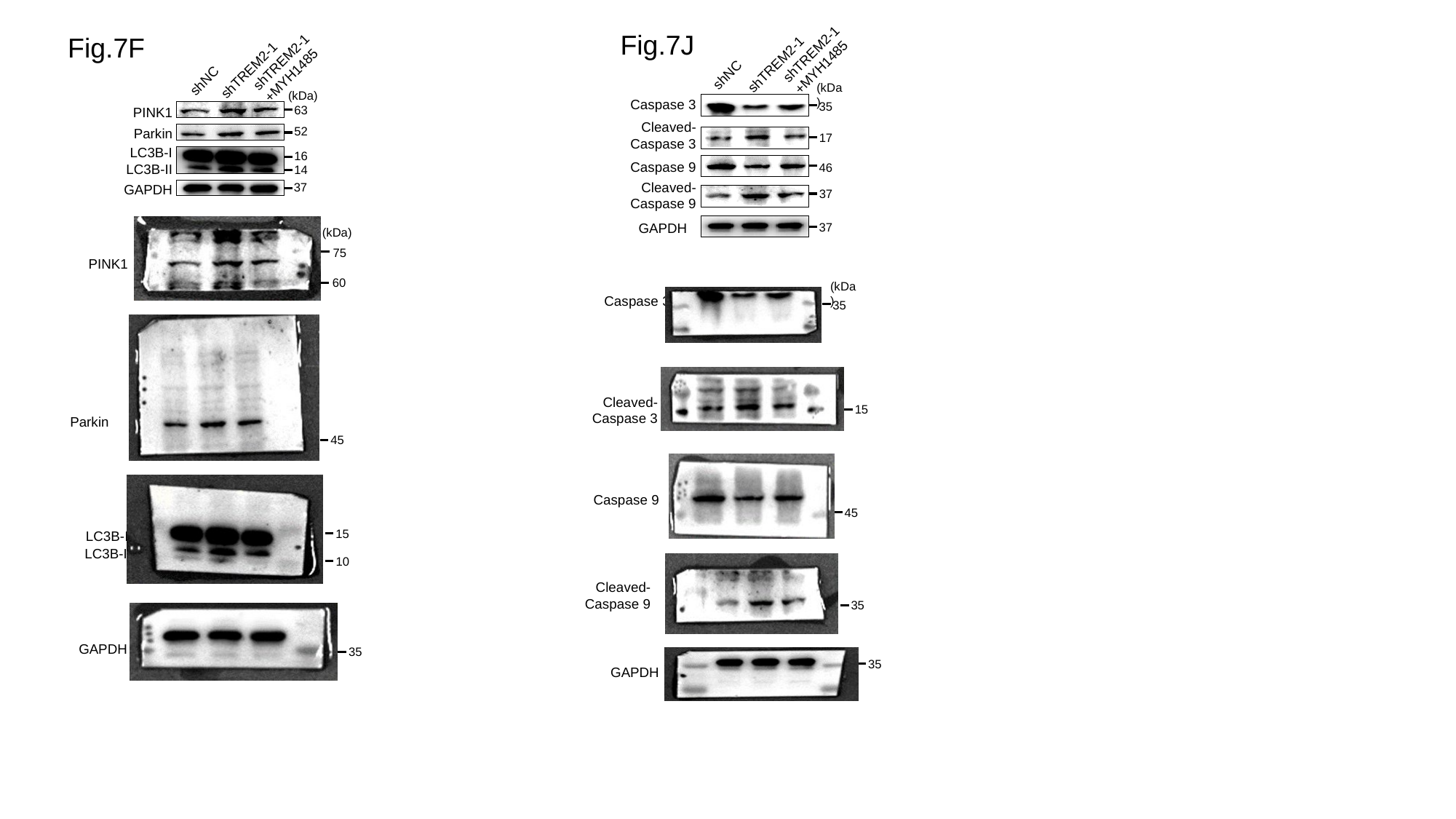

Fig.7J
Fig.7F
shTREM2-1
+MYH1485
shTREM2-1
+MYH1485
shTREM2-1
shTREM2-1
shNC
shNC
(kDa)
(kDa)
Caspase 3
35
63
PINK1
Cleaved-
Caspase 3
52
Parkin
17
LC3B-I
16
Caspase 9
46
LC3B-II
14
Cleaved-
Caspase 9
37
GAPDH
37
GAPDH
37
(kDa)
75
PINK1
60
(kDa)
Caspase 3
35
Cleaved-
Caspase 3
15
Parkin
45
Caspase 9
45
15
LC3B-I
LC3B-II
10
Cleaved-
Caspase 9
35
GAPDH
35
35
GAPDH

## Slide 7
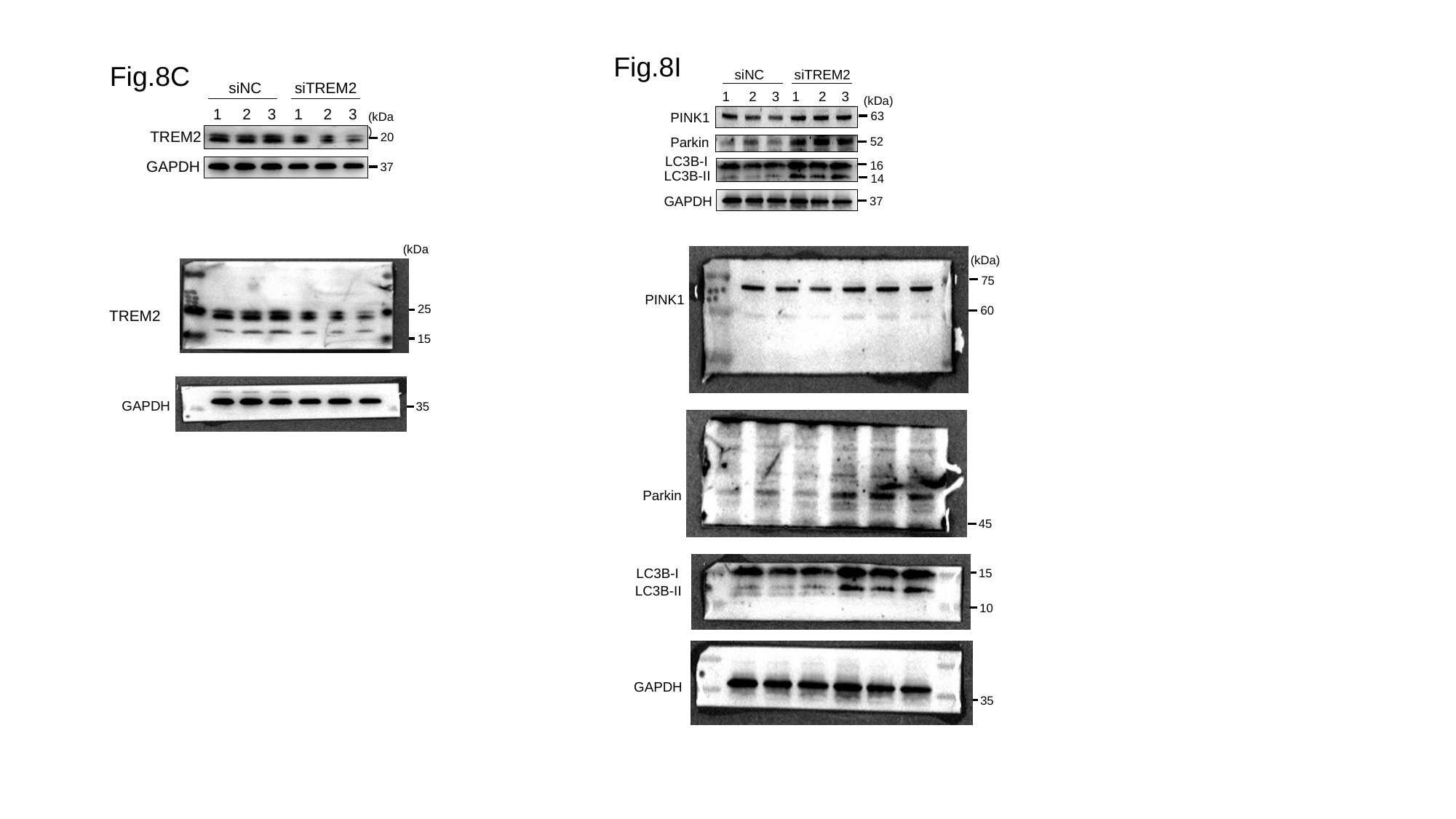

Fig.8I
Fig.8C
siNC
siTREM2
1 2 3
1 2 3
(kDa)
63
PINK1
Parkin
52
LC3B-I
16
LC3B-II
14
GAPDH
37
siNC
siTREM2
1 2 3
1 2 3
(kDa)
TREM2
20
GAPDH
37
(kDa)
(kDa)
75
PINK1
25
60
TREM2
15
GAPDH
35
Parkin
45
LC3B-I
15
LC3B-II
10
GAPDH
35
